# Comparative and Phylogenetic Analyses of Mitochondrial Genomes in Carabidae (Coleoptera: Adephaga)

**DOI:** 10.1002/ece3.71707

**Published:** 2025-07-02

**Authors:** Pingzhou Zhu, Tianyou Zhao, Yufang Meng, Hongliang Shi, Hongbin Liang, Chunyan Yang, Fan Song, Jinhong Zhou, Weidong Huang

**Affiliations:** ^1^ State Key Laboratory of Agricultural and Forestry Biosecurity, MOA Key Lab of Pest Monitoring and Green Management, College of Plant Protection China Agricultural University Beijing China; ^2^ Yunnan Tobacco Company Yuxi China; ^3^ College of Forestry Beijing Forestry University Beijing China; ^4^ Key Laboratory of Zoological Systematics and Evolution, Institute of Zoology Chinese Academy of Sciences Beijing China; ^5^ Genome Center of Biodiversity, Kunming Institute of Zoology Chinese Academy of Science Kunming China; ^6^ Yunnan Key Laboratory of Biodiversity Information Kunming China; ^7^ Yunnan International Joint Center of Urban Biodiversity Kunming China

**Keywords:** ground beetles, Insecta, misidentifications, mitogenome, phylogeny

## Abstract

The ground beetle represents one of the largest families in Coleoptera and is the main object of various ecological studies. However, the phylogeny of Carabidae remains unresolved, with insufficient publicly available mitogenomic data and persistent issues of misidentifications. In this study, six complete mitochondrial genomes (mitogenomes) from the genus *Harpalus* are reported, ranging from 16,066 to 16,948 bp in length and containing 37 typical genes and a control region. Combined with previously reported mitogenomic data, we found all protein‐coding genes (PCGs) initiated with standard start codons ATN or TTG and ended with TAN or an incomplete stop codon single T. Evolutionary rate analysis (Ka/Ks) revealed *atp8* was the fastest‐evolving gene, whereas *cox1* was the slowest. Additionally, 13 cases of suspected misidentifications in public Carabidae mitogenomes from GenBank were identified, with potential corrections suggested. Bayesian inference and maximum likelihood approaches were used to infer the phylogenetic relationships within Carabidae based on 121 mitogenomes and various datasets. The results confirmed the distinct separation of Cicindelidae from Carabidae. Within Carabidae, most subfamilies were supported as monophyletic, except Licininae and Platyninae. However, inter‐subfamily relationships remain poorly resolved, with Carabinae and Nebriinae consistently occupying basal positions and Harpalinae s. l. positioned terminally across different topologies. Our results provided more insights into the phylogenetic relationships within Carabidae, while expanding mitogenomic and genomic data across carabid lineages is crucial to resolve the uncertain phylogenetic relationships within this important beetle clade.

## Graphical abstract

In this study, six complete mitochondrial genomes (mitogenomes) from the genus *Harpalus* are reported. Additionally, 13 cases of suspected misidentifications in public Carabidae mitogenomes from GenBank were identified, with potential corrections suggested. Bayesian inference and maximum likelihood approaches were used to infer the phylogenetic relationships within Carabidae based on 121 mitogenomes and various datasets.

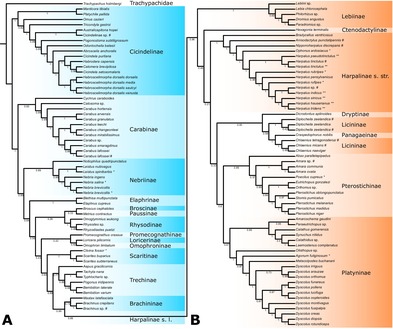

## 1 Introduction

The family Carabidae, or ground beetle, represents the third largest family of all living things, with approximately 41,000 described species distributed worldwide, only second to the family Staphylinidae (rove beetles, about 56,000 species) and Curculionidae (weevils, about 48,000 species) (Anichtchenko 2025; Lorenz 2025). While many ground beetle species retain fully developed hind wings and the ability to fly, the majority live predominantly on the ground, giving rise to their common name. The feeding habits of Carabidae are diverse, ranging from generalized carnivory to omnivory and even herbivory in a few species (Larochelle 1990). Due to their environmental sensitivity and the relative ease of collecting them, ground beetles are among the most extensively studied taxa in ecological research (Lövei and Sunderland 1996).

However, the phylogeny of the Carabidae family remains a subject of ongoing controversy, manifesting itself at three key levels. Firstly, whether the Carabidae clade encompasses Trachypachidae, Cicindelidae, Rhysodidae, and potentially other groups, as well as the precise phylogenetic relationships among these taxa has been a topic of active debate in recent years. This issue has largely reached a consensus, with evidence from UCEs, transcriptomic data, and morphological studies supporting the relationship Trachypachidae + (Carabidae + Cicindelidae), and affirming the inclusion of Rhysodinae within Carabidae (Baca et al. 2021; Beutel et al. 2020; Vasilikopoulos et al. 2021). Some studies on tiger beetles have also revealed the sister‐group relationship between tiger beetles and ground beetles (Duran and Gough 2020; Gough et al. 2020). Secondly, the relationships between the subfamilies of Carabidae, often referred to as the “basal carabids” (all subfamilies other than Harpalinae s. lat.), remain unresolved. Carabidae, representing over 90% of species within the Adephaga, are frequently sampled in broader studies of the suborder. Although these studies focus on inter‐family relationships within Adephaga, their phylogenetic trees still provide insights into relationships among sampled Carabidae subfamilies (Baca et al. 2021; Gustafson et al. 2020; López‐López and Vogler 2017; Vasilikopoulos et al. 2021). However, dedicated investigations into Carabidae subfamily phylogeny have seen little progress since Maddison et al. (1999). Thirdly, it is generally accepted that over half of all carabid species belong to the large monophyletic subfamily Harpalinae sensu lato. Within the modern taxonomic framework of Carabidae, some authors still adhere to a 32–34 subfamily classification system based on traditional views (Anichtchenko 2025; Lorenz 1998, 2005, 2025), while others have adopted a 15–22 subfamily arrangement by subsuming several apical subfamilies as supertribes or tribes under the expanded Harpalinae (Bouchard et al. 2011; Bousquet 2012; Löbl and Löbl 2017). Although the monophyly of most Harpalinae s. l. subfamilies, supertribes, and tribes has been confirmed, their precise phylogenetic relationships remain inconclusive (Ober 2002; Ober and Maddison 2008).

The insect mitochondrial genome (mitogenome) is a circular, double‐stranded DNA molecule, typically 14–20 kb in size, containing 37 highly conserved genes: 13 protein‐coding genes (PCGs), 22 transfer RNA genes (tRNAs), two ribosomal RNA genes (*rrnL* and *rrnS*) and an A + T‐rich control region (Boore 1999). As a compact and relatively stable genomic unit, the mitogenome has become an invaluable resource for studying evolution, phylogeny, population genetics, and taxonomy (Cameron 2014). With the advent of high‐throughput sequencing, research on mitogenomic structure, codon usage, and base composition across various insect taxa has expanded significantly, shedding light on their evolutionary and biogeographic patterns. More and more mitogenomes of Carabidae have been published (Bai, Ye, et al. 2022; Fang 2020; Kim et al. 2024), and some researchers have combined their sequencing data with publicly available datasets to construct phylogenetic trees encompassing a broader range of taxa (Lin et al. 2024) or to conduct detailed and specialized discussions (Kyndt and Kyndt 2022; Raupach et al. 2022). As of December 2024, more than 100 complete or nearly complete mitogenome sequences have been publicly available on GenBank, representing most lineages of basal carabids and the subfamily Harpalinae s. l. However, potential issues with species identification in some studies warrant attention, especially in an era of rapidly advancing molecular biology. Given the vast diversity of ground beetle species, additional mitogenomic data with accurate identification will greatly enhance our ability to explore and elucidate the origins and evolutionary history of this insect family.

The genus *Harpalus*, belonging to the subfamily Harpalinae and the tribe Harpalini, is one of the largest genera within the Carabidae. It includes over 400 species, primarily distributed across the Holarctic region, and is well‐known for its abundance, taxonomic complexity, and the challenges associated with species identification (Kataev 2023). During our investigation of the mitogenomes of Carabidae, we found some potential misidentifications in GenBank, and most of them were *Harpalus* species, such as *H. sinicus* (Yu et al. 2019), *H. anxius*, and *H. griseus* (Lin et al. 2024). To address this, we sequenced the mitochondrial genomes of six *Harpalus* species commonly found in Chinese agricultural fields and analyzed their genomic structure, base composition, AT content, and evolutionary rates, aiming to expand the mitogenomic database for this diverse genus and to rectify existing identification errors. Furthermore, to gain deeper insights into the mitogenomic characteristics and phylogeny of the Carabidae, we integrated our data with 114 previously published Carabidae mitogenomes and reconstructed the phylogenetic relationships within the family. This analysis provides new perspectives on the evolutionary relationships among the major subfamilies and tribes of Carabidae.

## 2 Materials and Methods

### 2.1 Sampling and DNA Extraction

Adult specimens of genus *Harpalus* were collected by Weidong Huang using the method of pitfall traps from agricultural fields in Yunnan Province, China (Table S1). All specimens were identified by Pingzhou Zhu based on morphological characters according to taxonomic literature (Kataev 2023; Kataev and Liang 2015). All specimens were preserved in 100% ethanol immediately in the field and stored at −20°C before DNA extraction. Total genomic DNA was extracted from the abdominal tissue using a DNeasy Blood and Tissue kit (QIAGEN) according to the manufacturer's protocol. The vouchers and DNA of the specimens are deposited at the College of Plant Protection, China Agricultural University, Beijing, China.

### 2.2 Sequencing and Bioinformatics Analyses

All Illumina TruSeq libraries were prepared with an average insert size of 350 bp and sequenced using the Illumina Novaseq 6000 platform (Berry Genomics, Beijing, China) with 150 bp paired‐end reads. High‐quality reads were utilized for mitogenome assembly with GetOrganelle v1.7.5.2 (Jin et al. 2020), using the animal database (‐F animal mt) as the parameter setting. Mitogenomes were annotated using MitoZ (Meng et al. 2019) with the invertebrate mitochondrial genetic code. The boundaries of the PCGs and rRNA genes were manually collated in Geneious Prime v11.0.18. Transfer RNA gene (tRNA) secondary structures were estimated using MITOS webservers (http://mitos2.bioinf.uni‐leipzig.de/index.py) (Donath et al. 2019).

To gain deeper insights into the mitogenomic characteristics and phylogeny of the Carabidae, 114 mitogenomes of Carabidae and one of Trachypachidae were downloaded from GenBank (Table S2). Nine of them were newly annotated from raw data using the method mentioned above. All these Carabidae mitogenomes were used for comparative mitogenomic analyses together with the six newly sequenced mitogenomes in the present study. The nucleotide composition, codon usage and relative synonymous codon usage (RSCU) of PCGs were calculated by MEGA 7.0 (Kumar et al. 2016). The rates of non‐synonymous substitution rate (Ka)/synonymous substitution rate (Ks) for each PCG was calculated by DnaSP 6.0 (Rozas et al. 2017). Composition skew analysis was performed using the AT‐skew = [A − T]/[A + T] and GC‐skew = [G − C]/[G + C] formulas (Perna and Kocher 1995).

### 2.3 Phylogenetic Analyses

Phylogenetic analyses were performed based on extensive taxon sampling consisting of all available 121 mitogenomes of Carabidae (six sequenced in this study and 114 downloaded from GenBank) and a Trachypachidae species selected as outgroup taxa (Table S2). PCGs and rRNAs were extracted, aligned, trimmed, and concatenated using PhyloSuite v1.2.2 (Zhang et al. 2020). The following three datasets were used to construct the corresponding phylogenetic trees: (1) Amino acid sequences of 13 PCGs (AA); (2) first and second codons of 13 PCGs combined with two rRNA genes (P12R); and (3) 13 PCGs combined with two rRNA genes (P123R).

The maximum likelihood (ML) analysis was conducted in IQTREE v2.1.2 (Minh et al. 2020). Substitution models were compared and selected according to the Bayesian information criterion (BIC) by using ModelFinder (Kalyaanamoorthy et al. 2017). An edge‐unlinked model was specified for both the full partition and the merged partition schemes. The reliability of the branching pattern was evaluated using bootstrap support (BS). A total of 1000 ultrafast bootstraps were used to evaluate the nodal support of the ML tree. Three partition schemes were applied: (1) no partition (NP); (2) full partition (FP) that provides the best‐fitting model for each individual gene; and (3) merged partition (MP) that implements a greedy strategy starting with the full partition model and subsequently merging pairs of genes until the model fit does not improve any further.

According to the user manual of PhyloBayes, the CAT‐GTR model consistently shows the best fit among all models implemented for the datasets exceeding 1000 aligned positions. Additionally, the CAT‐GTR model is likely a highly effective general‐purpose model for amino acid, DNA and RNA. Bayesian inferences (BI) was implemented under the CAT‐GTR model, using PhyloBayes‐MPI v1.8 (Lartillot et al. 2013; Lartillot and Philippe 2004, 2006). Two independent Markov Chain Monte Carlo (MCMC) runs of 5000 generations each were executed. Convergence was evaluated with the “bpcomp” and “tracecomp” procedure in the PhyloBayes package with a burn‐in of first 20% by the recommended criterion of maximum discrepancy < 0.1.

For ML analysis, all three datasets and all three partition schemes were used to construct nine ML trees. For BI analysis, only P123R dataset and NP partition scheme were used to construct one BI tree. The resulting phylogenetic trees were visualized in ITOL (Letunic and Bork, 2021).

## 3 Result

### 3.1 Mitogenome Organization and Base Composition

**FIGURE 1:**
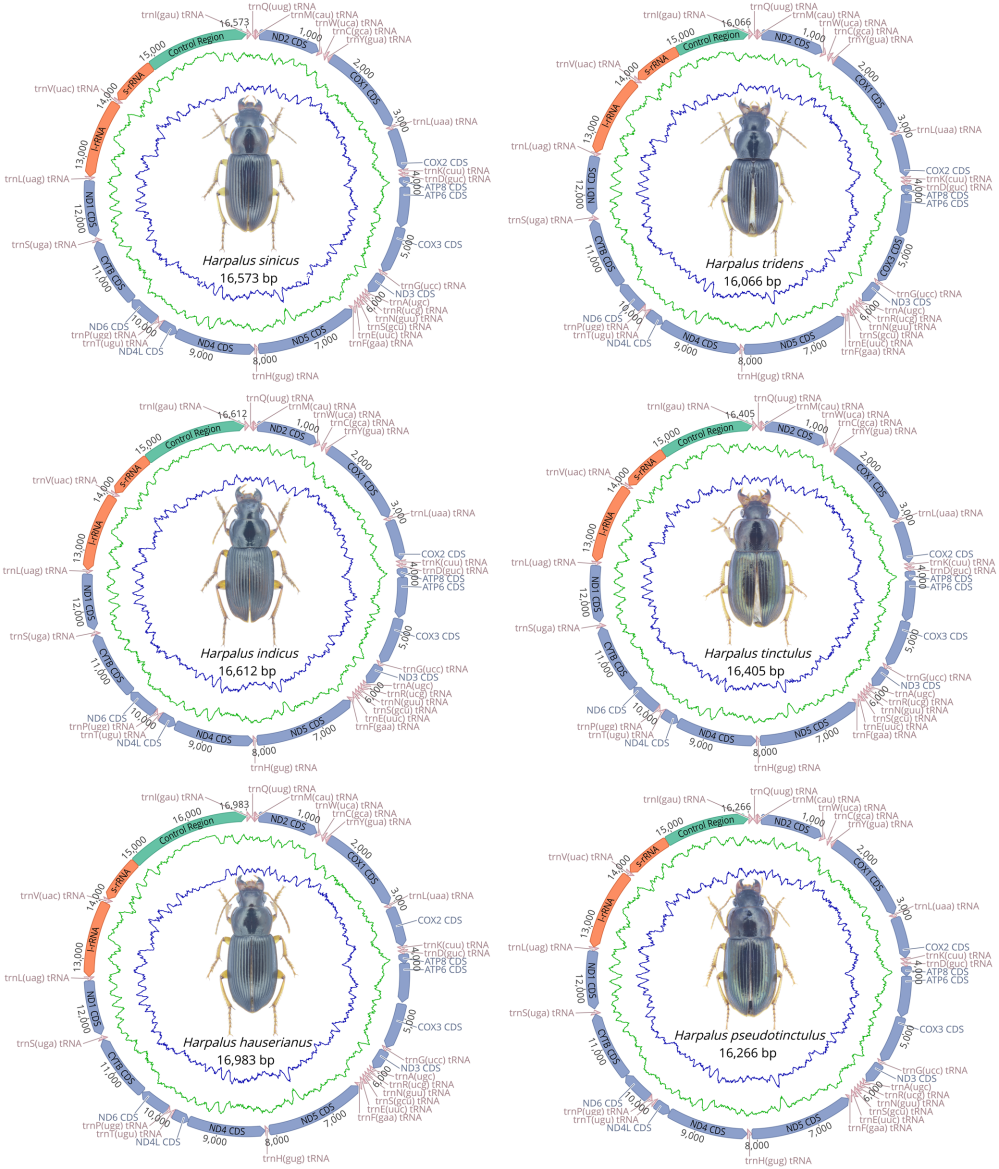
Circular maps of the six newly sequenced mitogenomes of Carabidae. Protein‐coding, ribosomal, and transfer RNA genes are indicated with standard abbreviations. Gene orientations are indicated by arrow directions. Protein‐coding genes, transfer RNA genes, control regions, and two ribosomal RNA genes are shown in yellow, purple, gray, and red, respectively.

All six newly sequenced mitogenomes are circular, double‐stranded molecules, and the range is from 16,066 bp ( *H. tridens* ) to 16,983 bp (*H. hauserianus*) with each consisting of 37 genes, including 13 PCGs, 22 tRNAs, two rRNAs, and a large noncoding region known as the control region (Figure 1, Table S3). The following accession numbers were assigned to the GenBank data for the six newly sequenced species: PV167288–PV167293. The 23 genes, including 9 PCGs and 14 tRNAs, are located on the major strand (J‐strand), and the other 14 genes (4 PCGs, 8 tRNAs, and 2 rRNAs) are located on the minor strand (N‐strand). The gene arrangement and orientation are similar to the typical mitogenomes of Coleoptera (Guo et al. 2023; Huang et al. 2023; Liu et al. 2025).

Base composition analysis revealed that whole sequences displayed a considerable bias toward adenine (A) and thymine (T), with AT content ranging from 79.6% to 80.4%. In addition, the PCGs, rRNAs, tRNAs, and control regions were all biased in nucleotide composition ((A + T)% > (G + C)%), which was congruent with most previously reported insect mitogenomes (Huang et al. 2025; Xu et al. 2025). Skew metrics of six mitogenomes showed positive AT‐skew in whole genomes, tRNAs, and control regions and positive GC‐skew in PCGs, rRNAs, and tRNAs (Table S4). During the process of replication and transcription, the asymmetries of nucleotide composition are generally regarded as an indicator for the direction of gene expression and the direction of replication (Wei et al. 2010).

### 3.2 Analyses of PCGs, tRNAs and rRNAs

The 13 PCGs of six newly sequenced mitogenomes range from 11,205 bp to 11,216 bp, which are typical for Coleoptera (Guo et al. 2023; Huang et al. 2023). Regarding start and stop codons, most PCGs use the conventional ATN (ATA/T/G) as the start codon (Table S3). However, in *nad1*, the unconventional TTG is consistently found. TAA or TAG is employed as stop codon in most PCGs, but the incomplete termination codon T is consistently used in *nad5* of all six species and *nad4l* of one species ( *H. indicus* ). Incomplete termination codons are commonly recognized across arthropod mitogenomes, which may be the result of mRNA maturation‐related posttranscriptional modification (Ojala et al. 1981).

**FIGURE 2:**
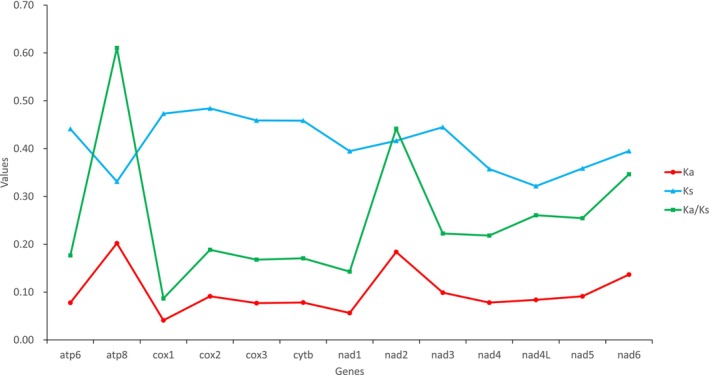
The Ka/Ks ratios of 13 protein‐coding genes. Ka is the nonsynonymous substitution rate, and Ks is the synonymous substitution rate.

**FIGURE 3:**
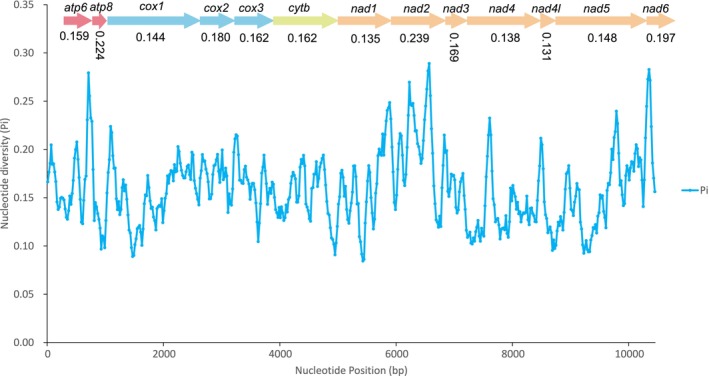
Sliding window analysis of 13 protein‐coding genes based on 114 Carabidae species. The blue line shows the value of nucleotide diversity Pi (window size = 100 bp, step size =20 bp).

Nucleotide diversity and the ratio of Ka/Ks across all carabid mitogenomes were calculated for each of the 13 PCGs to define their evolutionary patterns. The Ka/Ks value for *atp8* was the highest, followed by the *nad2*, *nad6*, *nad4l*, and the lowest value was for *cox1* (Figure 2) as that of nucleotide diversity (Figure 3). These findings suggested that *cox1* might serve as a possible marker for species identification because it was the least variable (Hebert et al. 2003; Huang et al. 2020). The results of these analyses suggest that the *atp8* is under the least selection pressure and the most fast‐evolving gene among the mitochondrial PCGs in Carabidae.

**FIGURE 4:**
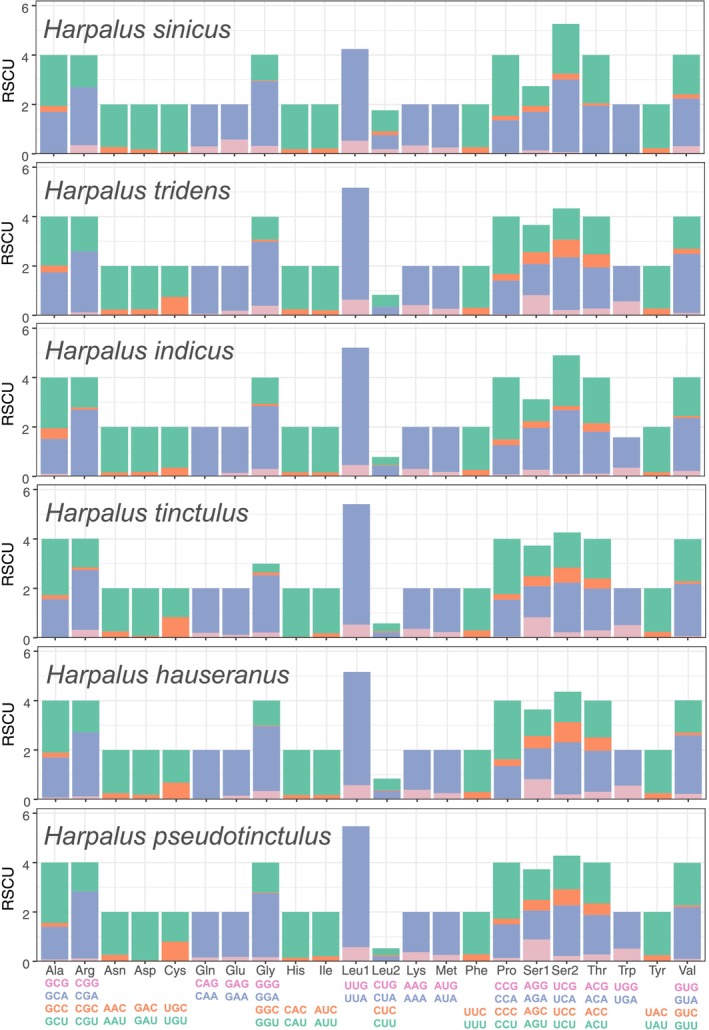
Relative synonymous codon usage in the protein‐coding genes of the six newly sequenced mitogenomes of Carabidae. Codon families are indicated below the *x*‐axis. The color of the codon family below the *x*‐axis corresponds to the color above the *x*‐axis. The stop codon is not given.

The RSCU in 13 PCGs was estimated for our six newly sequenced mitogenomes, as shown in Figure 4. Codon usage was similar among the sequenced Carabidae mitogenomes, with Leu, Ile, Met, Phe, and Ser being the five most frequently used amino acids. The majority of codons in all amino acids solely involved A or T (Figure 4), which was indicative of the high A + T content of PCGs.

For all newly sequenced Carabidae mitogenomes, the typical 22 tRNAs are expectedly recognized, and exhibit a typical clover‐leaf secondary structure, while *trnS1* lacks the DHU arm, a trait generally present in all Coleoptera insects as well as in other metazoan mitogenomes (Guo et al. 2023; Huang et al. 2023; Zhao et al. 2025; Figure S11). Both of rRNA genes, *rrnS* and *rrnL*, are located between *trnV* and the control region and between *trnV* and *trnL1*, respectively (Table S3).

### 3.3 Misidentifications in GenBank

**FIGURE 5:**
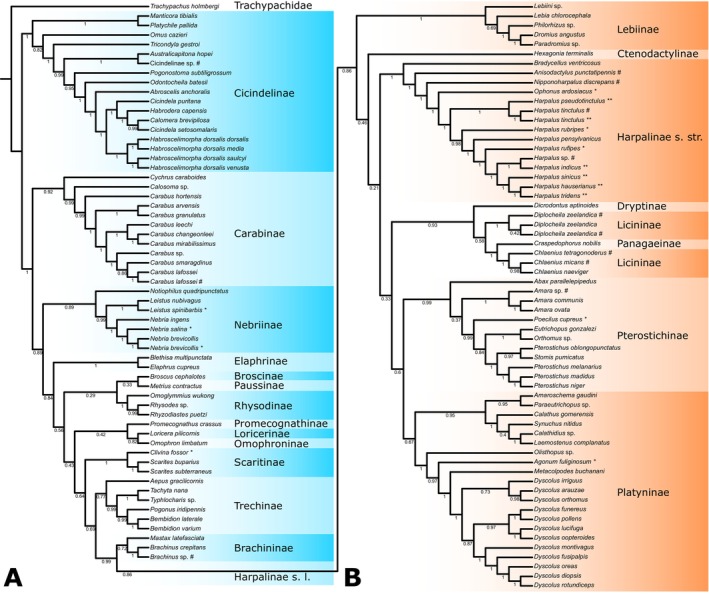
Phylogenetic trees of Carabidae inferred using Bayesian inference based on the P123R dataset and NP partition scheme. The numbers at the nodes are bootstrap values. Species names followed by an asterisk (*) are newly annotated in this study; those followed by a double asterisk (**) are newly sequenced and annotated in this study, and those followed by a hash symbol (#) are corrected names for species that were suspected to be misidentified. (A) Carabidae, with Harpalinae s. l. collapsed; (B) Harpalinae s. l.

Based on the locations in the phylogenetic trees (see the phylogenetic results below), *COI* barcoding, and other information in the original literature, such as images and collecting locations, several suspected misidentifications were discovered in GenBank (Table 1). Some misidentifications are due to morphological similarities, often involving confusion among closely related species within the same genus, but sometimes even occurring between different tribes or subfamilies. Others may result from the uploader accidentally submitting incorrect sequences. We have provided the correct species names for these suspected misidentifications based on our taxonomic knowledge of Carabidae. In the BI tree below (Figure 5), these species are shown with their corrected names, but in the ML trees of Figures S1–S10, they are still displayed with their original names.

**TABLE 1:** List of suspected misidentifications of Carabidae mitogenomes in GenBank.

GenBank No.	Species at present	Species suggested	References
MW629557.1	*Pheropsophus occipitalis*	*Carabus lafossei*	Ke et al. (2022)
MG253281.1	*Selenophorus alternans*	Cicindelinae sp.	Direct Submission
ON920164.1	*Galerita orientalis*	*Brachinus* sp.	Bai, Yang, et al. (2022)
ON929899.1	*Harpalus* sp.1	*Harpalus tinctulus*	Lin et al. (2024)
OP161482.1	*Harpalus* sp.2	*Nipponoharpalus discrepans*	Lin et al. (2024)
OP133272.1	*Harpalus griseus*	*Harpalus* sp.	Lin et al. (2024)
MN310888.1	*Harpalus sinicus*	*Anisodactylus punctatipennis*	Yu et al. (2019)
MT554376.1	*Chlaenius bioculatus*	*Chlaenius micans*	Direct Submission
OR536810.1	*Chlaenius bimaculatus*	*Chlaenius tetragonoderus*	Li et al. (2024)
MN995217.1	*Diplocheila zealandica*	*Diplocheila zeelandica*	Fang (2020)
MT554377.1	*Pterostichus prattii*	*Diplocheila zeelandica*	Direct Submission
MN335930.1	*Amara aulica*	*Amara* sp.	Li et al. (2020)

#### 3.3.1 MW629557.1: *Pheropsophus occipitalis*

In all the phylogenetic trees we constructed, this species is grouped together with *Carabus lafossei*, as well as other *Carabus* species. Subsequent BLAST analysis in GenBank showed that the *COI* barcoding of this species was highly consistent with *C. lafossei*, with similarities of 99.55%. In the original literature, the authors constructed phylogenetic trees based on the *COI* gene, which showed that this species did indeed cluster with some other species of *Pheropsophus* genus (Ke et al. 2022). But strangely, the authors used a *COI* gene fragments already in GenBank: MN264255, rather than their own newly sequenced ones. In fact, the appearances of *C. lafossei* and *P. occipitalis* are so distinct that even people with no basic entomological knowledge can easily distinguish them, and the authors have also discussed some morphological characteristics of the genus *Pheropsophus*. But the sequence MW629557.1 in GenBank actually shows that this is *C. lafossei*. Perhaps the authors sequenced the correct *P. occipitalis* and just accidentally uploaded the wrong sequence to GenBank.

#### 3.3.2 MG253281.1: *Selenophorus alternans*

This species and sequence are highly unusual. *Selenophorus alternans* , a member of Harpalinae s. str., is consistently clustered with *Australicapitona hopei* from Cicindelinae, with high support values across all phylogenetic trees. In the BLAST analysis of *COI* barcoding on GenBank, this species did not exhibit high similarity to any carabid beetle. The closest matches were *Blethisa multipunctata* , *Bradycellus ruficollis*, and several species of the genus *Agonum*, with a maximum similarity of only about 85%. However, mitogenome comparisons revealed that *Selenophorus alternans* indeed showed higher similarity to species within Cicindelinae, typically exceeding 82%. Among these, *Australicapitona hopei* exhibited the highest similarity at 87.10%, while most species, including those in Harpalinae s. str., showed similarity levels of approximately 80% or lower. Morphologically, *Selenophorus alternans* conforms to the general characteristics of typical Harpalinae and bears no resemblance to any Cicindelinae species. In any case, this molecular sequence is indeed closer to Cicindelinae than to other Carabidae, so we tentatively designate it as Cicindelinae sp.

#### 3.3.3 ON920164.1/NC_066084.1: *Galerita orientalis*

There is a very obvious and definite error in the original literature: the species in the image is clearly *Brachinus* sp. rather than *Galerita orientalis* (Bai, Yang, et al. 2022). This species is also grouped together with *Brachinus crepitans* in all the phylogenetic trees inferred in our study. However, the results of BLAST analysis of *COI* barcoding in GenBank showed that *Brachinus hirsutus* had the highest similarity with this species of only 90.45% and other *Brachinus* species range from 88% to 91%. Such low similarity is clearly not the same species, so we can only say that if the mitogenomes sequenced and uploaded by Bai, Yang, et al. (2022) are both from the specimen in the photo, then NC_066084.1 is probably a species of *Brachinus*.

#### 3.3.4 ON929899.1/NC_066079.1: *Harpalus* sp.1/*Harpalus anxius*

These two numbers and names correspond to the same sequence, and the species name in the original literature is *Harpalus* sp.1 (Lin et al. 2024). The *COI* barcoding and mitogenome of this species are 100% and 99.70% similar to our newly sequenced *H. tinctulus* , respectively, and they are always clustered together in different phylogenetic trees. It is therefore likely that this species is *H. tinctulus* .

#### 3.3.5 OP161482.1/NC_066078.1: *Harpalus* sp.2/*Harpalus discrepans*

These two numbers and names correspond to the same sequence, and the species name in the original literature is *Harpalus* sp.2 (Lin et al. 2024). The *COI* barcoding of this species is 99.71% similar to *H. discrepans* (HM180603.1). In fact, the “organism” column of this sequence has been correctly changed to *Nipponoharpalus discrepans* in GenBank, but the name is still wrongly *H. discrepans* . The monotypic taxon *Nipponoharpalus* Habu, 1973, described as a subgenus of *Harpalus*, is excluded from *Harpalus* and treated as a separate genus related to *Trichotichnus* now (Kataev 2023). Therefore, *N. discrepans* is the right combination in the current taxonomy system of Harpalini.

#### 3.3.6 OP133272.1/NC_066080.1: *Harpalus griseus*

The results of BLAST analysis of *COI* barcoding showed that *Harpalus griseus* had the highest similarity with this species of 97.23%–97.55% (different individuals in GenBank) and many other *Harpalus* species range from 91% to 95%. It is not similar enough to prove they are the same species, but probably the same genus. Therefore, it is recommended that this species be identified as *Harpalus* sp. rather than *H. griseus* .

#### 3.3.7 MN310888.1/NC_045094.1: *Harpalus sinicus*

The results of the BLAST analysis of *COI* barcoding showed that *Anisodactylus punctatipennis* had the highest similarity with this species of 99.34%–99.51% (different individuals in GenBank and our unpublished data). So, this species is likely *A. punctatipennis*. The two genera do resemble each other in appearance.

#### 3.3.8 MT554376.1: *Chlaenius bioculatus*

In China, many species of the genus *Chlaenius* exhibit two yellow spots on their elytra. Many of these species are common, sympatrically distributed, and difficult to identify, yet they may be distantly related, belong to different subgenera, or present taxonomic issues. The species mentioned in this and the following paragraph fall into this category, where misidentification is unsurprising. Based on BLAST analysis of *COI* barcodes on GenBank and our unpublished data, this species shows 100% similarity to *C. micans* , a very common *Chlaenius* species with two yellow spots in China. Additionally, while *C. bioculatus* is currently classified under the subgenus *Ocybatus* and *C. micans* under the subgenus *Achlaenius*, both may need to be reassigned to the subgenus *Lissauchenius* in our future taxonomic revisions.

#### 3.3.9 OR536810.1: *Chlaenius bimaculatus*

In the original literature, this species was identified as *Chlaenius bimaculatus* (Li et al. 2024), but in GenBank, it is listed as a subspecies of another species: *C. rufifemoratus bimaculatus*. They both belong to the subgenus *Lissauchenius* and are associated with numerous unresolved taxonomic issues. The image provided by the original literature allow for a tentative identification as *C*. (*Lissauchenius*) *tetragonoderus*, then confirmed by our unpublished molecular data. Incidentally, the specimen from Fujian also represents a new record of *C. tetragonoderus* for Fujian Province, China. By the way, the family name “Carabidae” in that literature was misspelled as “Carabidea” multiple times, even in the title.

#### 3.3.10 MN995217.1: *Diplocheila zealandica*

This is obviously a clerical error, as there is no such species named *Diplocheila zealandica*, and the correct spelling of the species name is *Diplocheila zeelandica*. It was also demonstrated by *COI* barcoding and results from all phylogenetic results. Very coincidentally, the two mitogenome sequences published consecutively have exactly the same length of 16,190 bp, although the collecting dates and locations are totally different (Fang 2020; Lin et al. 2024). At first, we suspected the same sequence was being used in both articles by some mistake, but after comparison, the two mitogenomes were 99.50% similar, and were indeed different individuals of the same species (confirmed by Xingyu Lin, personal communication).

#### 3.3.11 MT554377.1: *Pterostichus prattii*

This is another obvious misidentification, easily confirmed by molecular data and identical to the previous species. It is interesting that although the genus *Pterostichus* and *Diplocheila* are quite distantly related, these two species do bear some resemblance in their outward appearance. They are both black in color and have similar sizes and shapes of the pronotum, so it is understandable that a lay person might confuse them.

#### 3.3.12 MN335930.1: *Amara aulica*

*Amara aulica* is mainly distributed in the western and northern parts of the Palearctic Region and has invaded the Nearctic Region. In China, this species is only found in Xinjiang and Northeast regions, while the original literature mentions that this specimen was collected from Henan, a central province, which is likely a misidentification (Li et al. 2020). The results of BLAST analysis of *COI* barcoding showed that *A. gigantea* had the highest similarity with this species of 98.14% and many other *Amara* species range from 88% to 98% ( *A. aulica* of which about 92%). It is not similar enough to prove they are the same species, but probably the same genus. Therefore, it is recommended that this species be identified as *Amara* sp.

### 3.4 Phylogenetic Relationships

#### 3.4.1 ML Trees

Based on three different datasets (P123R, P12R, and AA) and three distinct partitioning strategies (FP, MP, and NP), nine ML trees were constructed (Figure S1–S9). There are substantial topological differences were revealed among the phylogenetic trees.

For basal carabids, the monophyly of most subfamilies was strongly supported, except for the followings. In the three AA trees, *Notiophilus* was separated from other Nebriinae and even grouped within the Carabinae lineage. Scaritinae appeared polyphyletic in the P12R/NP trees. Except for being monophyletic in the P123R/NP tree, Trechinae was paraphyletic in all other eight trees, consistently including *Mastax* + Rhysodinae, and sometimes Omophroninae. Brachininae was polyphyletic in all trees, with *Mastax* consistently clustering with Rhysodinae, while *Brachinus* sometimes grouped with Dryptinae to form a sister group to other Harpalinae s. l. (in P123R and P12R) and sometimes acted alone as the sister group to Harpalinae s. l. (in AA). The phylogenetic relationships among subfamilies exhibit significant topological differences across trees, and the support values for most subfamily nodes are below 95. Only Nebriinae and/or Carabinae are consistently positioned at the basal branches, while Cicindelidae is almost always nested within Carabidae (except in P12R/FP analyses). Harpalinae s. l. is consistently placed at the terminal branches.

The monophyly of Harpalinae s. l. is strongly supported, but it is consistently associated with the insertion of Brachinus. Within Harpalinae s. l., Panagaeinae always nested within Licininae, Platyninae was often polyphyletic, while Lebiinae was occasionally paraphyletic. The monophyly of the remaining subfamilies was generally well‐supported, except for Pterostichinae, which sometimes showed low support. Relationships among the subfamilies varied greatly across different trees, and most nodes had low support values.

#### 3.4.2 BI Tree

The results of the BI tree differ from most ML trees (Figure 5; Figure S10). Nearly all subfamilies are monophyletic, including Trechinae and Brachininae, which exhibit considerable issues in the ML trees. Cicindelidae occupies the most basal position in the phylogenetic tree, followed by Carabinae, Nebriinae, and Elaphrinae, all with relatively high support values.

Within Harpalinae s. l., Brachininae remains nested but does not show a close relationship with Dryptinae. Panagaeinae remains embedded within Licininae. Except for the relatively low support for Platyninae, the monophyly of other subfamilies is strongly supported. However, key nodes defining inter‐subfamily relationships continue to exhibit low support.

## 4 Discussion

### 4.1 Carabidae, Trachypachidae, and Cicindelidae

Previous studies based on gene fragments or mitogenomes have suggested that Trachypachidae is nested within Carabidae (Hunt et al. 2007; Lin et al. 2024; Maddison et al. 2009; Raupach et al. 2022; Timmermans et al. 2015) or formed a sister group with Cicindelidae (López‐López and Vogler 2017). However, the hypothesis of Trachypachidae as the sister group to (Carabidae + Cicindelidae), supported by UCEs, transcriptomes, and morphological data, is more convincing and has gained near‐universal acceptance (Baca et al. 2021; Beutel et al. 2020; Vasilikopoulos et al. 2021). In our study, when outgroups included aquatic adephagan taxa such as Gyrinidae and Haliplidae, conclusions similar to earlier research were obtained (e.g., Trachypachidae nested within Carabidae, the unusual placement of Rhysodinae, etc.). This discrepancy may stem from the limited data capacity of mitogenomes compared to extensive nuclear datasets or the unique evolutionary history of mitochondrial DNA. Given that more reliable phylogenetic relationships within Adephaga have already been established, we selected only Trachypachidae as the outgroup, temporarily avoiding potential issues associated with phylogenetic analyses based on mitogenomes. The discordance between mitochondrial and nuclear phylogenetic signals will be studied in our future work.

When Trachypachidae was designated as the sole outgroup, the Cicindelidae formed a sister group to the rest of the Carabidae in BI tree and one ML tree (P12R/FP), while in all other ML trees, they were nested within the Carabidae. This further reflects the issue of inconsistent phylogenetic signals between mitochondrial genomes and nuclear genes, while also highlighting that BI tree may work better than ML trees for this problem.

### 4.2 Carabinae and Nebriinae

These two subfamilies have been the subject of some excellent research, including morphology (Deuve 2019; Ledoux and Roux 2005) and molecular studies (Kavanaugh et al. 2021; Sota et al. 2022). Morphologically, both subfamilies exhibit several plesiomorphic traits, such as open procoxal cavities and apical placement of both spurs on the fore tibiae. Phylogenetically, they are often recovered as basal lineages within Carabidae in molecular studies. In most previous research, these subfamilies have been consistently demonstrated to be monophyletic. However, a phylogenetic study based on mitogenome revealed that *Cychrus* (Carabinae) grouped with *Notiophilus* (Nebriinae), rather than other Carabinae members (Lin et al. 2024). In the present study, a similar result was observed only in the AA/NP tree, whereas the monophyly of Carabinae was strongly supported in all other trees. Additionally, *Notiophilus* did not group with other Nebriinae members in the AA/FP and AA/MP trees, suggesting that nucleotide sequences may be more suitable than amino acid sequences for resolving the phylogenetic relationships within Carabinae and Nebriinae.

Regarding inter‐subfamily relationships, our results align with previous findings. The sister‐group relationship between these two subfamilies was supported in all three P123R trees (Vasilikopoulos et al. 2021) but was rejected in BI tree and all three P12R trees (Raupach et al. 2022).

### 4.3 Trechinae, Rhysodinae, and Brachininae

There has been a comprehensive study of molecular systematics based on gene fragments of Trechinae (Maddison et al. 2019). However, in this study, Trechinae is recovered as monophyletic only in the BI tree and P123R/NP tree, and even then, with very low support. In all other eight trees, Trechinae is paraphyletic, consistently including *Mastax* and Rhysodinae, and occasionally Omophroninae.

Rhysodinae, comprising approximately 400 species with highly distinctive morphology, typically inhabits decaying wood (Wang 2020). Its placement within Carabidae has been debated for a long time, but it is now generally treated as a subfamily of Carabidae. In our study, the monophyly of Rhysodinae is strongly supported, and it displays extraordinarily long branch lengths in the phylogenetic trees, consistent with previous research. This phenomenon is attributed to a high rate of nonsynonymous nucleotide substitutions, which may reflect strong positive selection pressure on this group.

Brachininae, commonly known as bombardier beetle, is famous for its ability to eject high‐temperature defensive chemicals in an explosive manner (Di Giulio et al. 2015). Its unique chemical defense mechanism, coupled with a more flexible abdomen (with 8 visible ventrites in males and 7 in females, rather than the typical 6), strongly supports the monophyly of Brachininae. However, paradoxically, despite its morphological and biochemical distinctiveness, molecular studies usually indicate that Brachininae is non‐monophyletic (Maddison et al. 1999; Ober 2002). Previous studies have frequently placed Brachininae either as a sister group to Harpalinae s. l. or embedded within its basal lineages. Our study supports this pattern, with Brachininae being monophyletic in BI tree but polyphyletic in all ML trees. Specifically, *Mastax* consistently clustered with Rhysodinae, while *Brachinus* sometimes grouped with Dryptinae to form a sister group to other Harpalinae s. l. (in P123R and P12R) and sometimes acted alone as the sister group to Harpalinae s. l. (in AA).

The paraphyly of Trechinae is highly unexpected, and even more surprising is the sister‐group relationship between *Mastax* and Rhysodinae, a result absent from all previous studies. We hypothesize this is caused by long‐branch attraction. In addition to the well‐known long branches of Rhysodinae, *Mastax* and some Trechinae lineages also exhibit relatively long branches, leading to their clustering in the ML trees. BI effectively resolves this issue, and the results of the BI tree likely reflect a more accurate depiction of phylogenetic relationships.

### 4.4 Others Basal Carabids

In this study, several other basal carabid subfamilies were also included, such as Elaphrinae, Promecognathinae, Broscinae, Omophroninae, Loricerinae, Scaritinae, and Paussinae. However, due to the limited sampling of these subfamilies, the monophyly of each could not be meaningfully assessed. Furthermore, the support values for the nodes connecting these subfamilies were consistently low, leaving their phylogenetic relationships entirely unresolved.

### 4.5 Harpalinae s. l.

Harpalinae s. l. is one of the most successful radiations within the Carabidae family, accounting for a significant proportion of extant carabid species, and is typically considered monophyletic, occupying the most derived branches of the phylogenetic tree. In this study, the monophyly of Harpalinae s. l. was not well‐supported due to the inclusion of *Brachinus*, although the node representing Harpalinae s. l. including *Brachinus* consistently received high support. The monophyly of most clades within Harpalinae s. l. was strongly supported, yet the relationships between them remain unresolved.

### 4.6 Lebiinae

The subfamily Lebiinae represents a highly successful lineage of arboreal‐adapted carabid beetles. Based on the characteristic truncate elytra, Lebiinae is sometimes discussed alongside other groups with similar traits under the term Lebiomorph assemblage (Ober and Maddison 2008) or the older designation Truncatipennes (Habu 1967; Jedlička 1963). Previous studies have indicated that the Lebiomorph assemblage and even the tribe Lebiini are nonmonophyletic.

In this study, due to extremely limited sampling that included only a few representatives of Lebiini, the monophyly of Lebiinae was strongly supported in the BI, P123R, and P12R trees but received weak support (FP) or was inferred as paraphyletic (MP and NP) in the AA trees. Such sparse sampling is clearly insufficient to represent the entirety of Lebiinae. Future studies must incorporate a broader array of lineages and species to draw more robust conclusions.

### 4.7 Harpalinae s. str

In this study, the results for Harpalinae s. str. were highly robust, with nearly identical topologies with high support values across all phylogenetic trees. At the tribal level, the relationship was resolved as Stenolophini + (Anisodactylini + Harpalini). At the generic level, the topology was resolved as genus *Nipponoharpalus* + ((genus *Ophonus* + (subgenus *Harpalus* + subgenus *Zangoharpalus*)) + subgenus *Pseudoophonus*) (ML trees) or genus *Nipponoharpalus* + (genus *Ophonus* + ((subgenus *Harpalus* + subgenus *Zangoharpalus*) + subgenus *Pseudoophonus*)) (BI tree). The above relationships were strongly supported in the corresponding trees, except for three P12R trees, where genus *Ophonus* and subgenus *Harpalus* formed a sister group with lower support.

Previous studies based on *COI* gene fragments revealed that most tribal (or subtribal) ranks were non‐monophyletic and phylogenetic relationships remained unresolved (Martínez‐Navarro et al. 2005). In contrast, our results showed that all tribes were monophyletic with well‐resolved relationships, although this may due to the limited sampling in our study. The generic status of *Nipponoharpalus* was confirmed, but that of *Ophonus* was not, which can be distinguished from *Harpalus* mainly by the pubescent head. If the identification of *Ophonus ardosiacus* is accurate, further studies are needed to verify the generic status of *Ophonus*. Additionally, subgenus *Harpalus* and subgenus *Zangoharpalus* are more closely related to each other than to subgenus *Pseudoophonus*, consistent with the relationships inferred from morphological keys (Kataev 2023).

### 4.8 Licininae and Panagaeinae

A distinctive synapomorphy, the lateral attachment of the terminal segment of the labial and maxillary palps to the penultimate segment, strongly supports the monophyly of Panagaeinae. While members of Licininae share few morphological synapomorphies.

Previous studies revealed that Oodini, Chlaeniini, and Panagaeini share crossed epipleura and secrete methacrylic acid (Oodini) or phenol (Chlaeniini and Panagaeini) through their pygidial glands. In contrast, Licinini has non‐crossed epipleura and secretes formic acid as the primary component of its defensive glands (Bousquet 2012). Despite their similarities in male protarsi, genitalia, and larvae, molecular data indicate that Licinini is unrelated to the other groups (Ober and Maddison 2008).

In this study, the clade Licinini + (Panagaeinae + Chlaenini) received strong support across all phylogenetic trees, with slightly lower support only in the NP/P123R trees. This represents one of the few highly reliable results obtained in this analysis. Based on this result, Chlaenini should be removed from Licininae, or Panagaeinae could be subsumed under Licininae. However, final taxonomic revisions will require more comprehensive studies incorporating critical groups, such as Oodini.

### 4.9 Pterostichinae

Pterostichinae represents another complex group within Harpalinae s. l. that lacks clear synapomorphic traits to support its monophyly. Currently, it is tentatively grouped based on the shared characteristic of a relatively robust body. In this study, only a few species from two tribes, Pterostichini and Zabrini, were sampled. These taxa consistently formed a clade in all phylogenetic trees, exhibiting relationships such as Pterostichini + Zabrini or Abax + (Pterostichini + Zabrini). However, high support values were consistently observed only for the Zabrini lineage. Pterostichinae as a whole received high support only in the BI tree and P12R trees, while Pterostichini displayed weak support (P12R and AA) or was resolved as paraphyletic (BI and P123R). The phylogenetic position of Pterostichinae remained unstable across the analyses and never clustered with Sphodrini in any tree, contrasting with previous findings (Ober and Maddison 2008; Vasilikopoulos et al. 2021). Furthermore, many significant groups within Pterostichinae, such as Cnemalobini and Morionini, are not sampled, and greater taxonomic representation is necessary to accurately resolve the subfamily's phylogenetic placement.

### 4.10 Platyninae

As shown in the results section, Platyninae is the only subfamily within Harpalinae s. l. which is probably polyphyletic in this study. This subfamily includes approximately 4000 species classified into three tribes: Omphreini (around 20 species, distributed in the Balkans), Sphodrini (around 1000 species, distributed across the Northern Hemisphere), and Platynini (around 3000 species, distributed worldwide). Among them, Platynini represents one of the most complex groups within Harpalinae s. l., known for its taxonomic ambiguity and notorious difficulty in identification.

In this study, the monophyly of both Sphodrini and Platynini was strongly supported individually, but the two tribes only grouped together in the BI, AA/MP and AA/NP trees with relatively low support. Previous studies have also suggested that Sphodrini is not closely related to Platynini (Ruiz et al. 2009) and may even share a closer relationship with Pterostichinae (Ober and Maddison 2008; Vasilikopoulos et al. 2021). Additionally, not only Platyninae, the monophyly of Platynini is also doubtful, for no morphological synapomorphies supporting it. We believe that Sphodrini is genuinely monophyletic, supported by the presence of glabrous basal gonocoxites in females. However, the apparent monophyly of Platynini is likely due to uneven sampling, with only four genera sampled out of approximately 180. Furthermore, the third tribe, Omphreini, was entirely unsampled, revealing significant limitations in this study concerning Platyninae.

On the other hand, identifying Platynini specimens is exceedingly challenging, even for professional carabid taxonomists, and even when restricted to the genus level. This difficulty further constrains the increase of sampling in molecular phylogenetic studies, as misidentified specimens are of no value. Consequently, the primary task for this group is revising and improving its taxonomic framework, starting with morphology and COI barcoding.

## 5 Conclusions

In this study, six mitogenomes of genus *Harpalus* are reported here for the first time. The composition and length of the genes of these new mitogenomes are the same as those of an ancestral insect mitogenome, and the selection pressure and polymorphism of each gene are consistent with those previously reported for carabids mitochondrial genes. According to the nonsynonymous/synonymous mutation ratio (Ka/Ks) of all PCGs, the highest and the lowest evolutionary rates are found for *atp8* and *cox1*, respectively. Among the public carabids mitogenomes in GenBank, 13 suspected misidentifications are discovered and possible actual species are suggested for each of them.

Coupled with published mitogenomic data, phylogenetic relationships within Carabidae were investigated based on various datasets and partition strategies. Our results confirm the separation of Cicindelidae and Carabidae. Among Carabidae, the monophyly of most subfamilies is well supported, except for Licininae and Platyninae. The phylogenetic relationships among the subfamilies remain poorly resolved. Only Carabinae and Nebriinae are commonly placed in more basal positions, Harpalinae s. l. is consistently found at the most derived position, and Brachininae is embedded within Harpalinae s.l., occupying the most basal position within it.

## Author Contributions

**Pingzhou Zhu:** conceptualization (equal), data curation (equal), formal analysis (equal), investigation (equal), visualization (equal), writing – original draft (equal), writing – review and editing (equal). **Tianyou Zhao:** data curation (equal), formal analysis (equal), methodology (equal), software (equal), visualization (equal), writing – review and editing (equal). **Yufang Meng:** funding acquisition (equal), project administration (equal), resources (equal). **Hongliang Shi:** resources (equal), supervision (equal), writing – review and editing (equal). **Hongbin Liang:** resources (equal), supervision (equal), writing – review and editing (equal). **Chunyan Yang:** funding acquisition (equal), methodology (equal), project administration (equal), resources (equal), supervision (equal). **Fan Song:** funding acquisition (equal), project administration (equal), resources (equal), visualization (equal), writing – review and editing (equal). **Jinhong Zhou:** funding acquisition (equal), project administration (equal), resources (equal). **Weidong Huang:** conceptualization (equal), data curation (equal), formal analysis (equal), investigation (equal), methodology (equal), software (equal), supervision (equal), visualization (equal), writing – review and editing (equal).

## Ethics Statement

The authors have nothing to report.

## Conflicts of Interest

The authors declare no conflicts of interest.

## Supporting information


Data S1:

## Data Availability

The data that supports the findings of this study are available in the Supporting Information S1 of this article. The datasets presented in this study can be found in online repositories and the study is deposited in the NCBI repository with accession numbers: PV167288–PV167293.
