# Supplementary material for: Comparative and Phylogenetic Analyses of Mitochondrial Genomes in Carabidae (Coleoptera: Adephaga)
Source: Ecol Evol. 2025 Jul 2;15(7):e71707. doi: 10.1002/ece3.71707 (PMC12222001; doi:10.1002/ece3.71707)
Supplement: Supplementary file 1 — Data S1: [file ECE3-15-e71707-s001.docx]

Table S1 The voucher information of the specimens sequenced in this study.

| No. | Species | Locality | Crops | Date |
| --- | --- | --- | --- | --- |
| CA136 | *Harpalus* (*Pseudoophonus*)  *sinicus* Hope, 1845 | Kunming City, Shilin County  24.7115°N, 103.2883°E, 1653 m | maize | 2022.V.13 |
| CA155 | *Harpalus* (*Pseudoophonus*)  *tridens* Morawitz, 1862 | Kunming City, Shilin County  24.7115°N, 103.2883°E, 1653 m | maize | 2022.V.12 |
| CA163 | *Harpalus* (*Pseudoophonus*)  *indicus orientalis* Kataev, 2014 | Baoshan City, Tengchong City  25.3986°N, 98.6329°E, 1476 m | tobacco | 2022.VI.30 |
| CA297 | *Harpalus* (*Zangoharpalus*)  *tinctulus luteicornoides* Breit, 1913 | Honghe Prefecture, Mile City  24.3772°N, 103.4599°E, 1415 m | tobacco | 2022.VI.7 |
| CA333 | *Harpalus* (*Pseudoophonus*)  *hauserianus* Schauberger, 1929 | Honghe Prefecture, Mile City  24.5354°N, 103.4046°E, 1774 m | tobacco | 2022.VIII.1 |
| CA484 | *Harpalus* (*Zangoharpalus*)  *pseudotinctulus* Schauberger, 1932 | Honghe Prefecture, Mile City  24.3747°N, 103.4600°E, 1412 m | tobacco | 2022.VII.2 |

Table S2 List of taxa analyzed in this study together with relevant information.

| GenBank No. | Subfamily/Family | Tribe | Species | Reference |
| --- | --- | --- | --- | --- |
| JX412826.1 | Brachininae | Brachinini | *Brachinus crepitans* | Direct Submission |
| NC_065263.1 | Brachininae | Brachinini | *Mastax latefasciata* | Bai et al, 2022b |
| MW629557.1 | Brachininae | Brachinini | *Pheropsophus occipitalis#* | Ke et al, 2022 |
| MF497819.1 | Broscinae | Broscini | *Broscus cephalotes* | López-López & Vogler 2017 |
| NC_018339.1 | Carabinae | Carabini | *Calosoma* sp. BYU-CO241 | Song et al, 2010 |
| MN122835.1 | Carabinae | Carabini | *Carabus arvensis* | Direct Submission |
| NC_046469.1 | Carabinae | Carabini | *Carabus changeonleei* | Wang et al, 2019 |
| NC_044759.1 | Carabinae | Carabini | *Carabus granulatus* | Direct Submission |
| MN122850.1 | Carabinae | Carabini | *Carabus hortensis* | Direct Submission |
| NC_036507.1 | Carabinae | Carabini | *Carabus lafossei* | Liu et al, 2018 |
| OQ621646.1 | Carabinae | Carabini | *Carabus leechi yooni* | Kwon et al, 2023 |
| NC_016469.1 | Carabinae | Carabini | *Carabus mirabilissimus mirabilissimus* | Wan et al, 2012 |
| MN480425.1 | Carabinae | Carabini | *Carabus smaragdinus* | Oh et al, 2020 |
| OP161481.1 | Carabinae | Carabini | *Carabus* sp. | Lin et al, 2024 |
| MT872703.1 | Carabinae | Carabini | *Cychrus caraboides* | Direct Submission |
| NC_038191.1 | Cicindelinae | Cicindelini | *Abroscelis anchoralis* | Wang et al, 2018 |
| NC_068073.1 | Cicindelinae | Cicindelini | *Calomera brevipilosa* | Direct Submission |
| MW442537.1 | Cicindelinae | Cicindelini | *Cicindela puritana* | Direct Submission |
| MT554379.1 | Cicindelinae | Cicindelini | *Cicindela setosomalaris* | Direct Submission |
| JX412824.1 | Cicindelinae | Cicindelini | *Habrodera capensis* | Direct Submission |
| MW074920.1 | Cicindelinae | Cicindelini | *Habroscelimorpha dorsalis dorsalis* | Direct Submission |
| MW113736.1 | Cicindelinae | Cicindelini | *Habroscelimorpha dorsalis media* | Direct Submission |
| MW450885.1 | Cicindelinae | Cicindelini | *Habroscelimorpha dorsalis saulcyi* | Direct Submission |
| MW450884.1 | Cicindelinae | Cicindelini | *Habroscelimorpha dorsalis venusta* | Direct Submission |
| MF497818.1 | Cicindelinae | Cicindelini | *Odontocheila batesii* | López-López & Vogler 2017 |
| MF497820.1 | Cicindelinae | Collyridini | *Pogonostoma subtiligrossum* | López-López & Vogler 2017 |
| MT554380.1 | Cicindelinae | Collyridini | *Tricondyla gestroi* | Direct Submission |
| MF497816.1 | Cicindelinae | Megacephalini | *Australicapitona hopei* | López-López & Vogler 2017 |
| NC_035714.1 | Cicindelinae | Platychilini | *Manticora tibialis* | López-López & Vogler 2017 |
| MF497813.1 | Cicindelinae | Platychilini | *Omus cazieri* | López-López & Vogler 2017 |
| MF497814.1 | Cicindelinae | Platychilini | *Platychile pallida* | López-López & Vogler 2017 |
| JX412768.1 | Ctenodactylinae | Hexagoniini | *Hexagonia terminalis* | Direct Submission |
| NC_066084.1 | Dryptinae | Galeritini | *Galerita orientalis#* | Bai et al, 2022a |
| OQ716336.1 | Dryptinae | Zuphiini | *Dicrodontus aptinoides* | Jiménez-García et al, 2023 |
| KX087243.1 | Elaphrinae | Elaphrini | *Blethisa multipunctata* | Direct Submission |
| KX087286.1 | Elaphrinae | Elaphrini | *Elaphrus cupreus* | Direct Submission |
| NC_066079.1 | Harpalinae | Harpalini | *Harpalus anxius#* | Lin et al, 2024 |
| NC_066080.1 | Harpalinae | Harpalini | *Harpalus griseus#* | Lin et al, 2024 |
| CA333 | Harpalinae | Harpalini | *Harpalus hauserianus*** | This study |
| CA163 | Harpalinae | Harpalini | *Harpalus indicus*** | This study |
| NC_046953.1 | Harpalinae | Harpalini | *Harpalus pensylvanicus* | Kieran, 2020 |
| CA484 | Harpalinae | Harpalini | *Harpalus pseudotinctulus*** | This study |
| OY720467 | Harpalinae | Harpalini | *Harpalus rubripes** | Direct Submission |
| OX596293 | Harpalinae | Harpalini | *Harpalus rufipes** | Direct Submission |
| NC_045094.1 | Harpalinae | Harpalini | *Harpalus sinicus#* | Yu et al, 2019 |
| CA136 | Harpalinae | Harpalini | *Harpalus sinicus*** | This study |
| CA297 | Harpalinae | Harpalini | *Harpalus tinctulus*** | This study |
| CA155 | Harpalinae | Harpalini | *Harpalus tridens*** | This study |
| NC_066078.1 | Harpalinae | Harpalini | *Harpalus discrepans#* | Lin et al, 2024 |
| OW964842 | Harpalinae | Harpalini | *Ophonus ardosiacus** | Direct Submission |
| MG253281.1 | Harpalinae | Harpalini | *Selenophorus alternans#* | Direct Submission |
| OQ716321.1 | Harpalinae | Stenolophini | *Bradycellus ventricosus* | Jiménez-García et al, 2023 |
| OQ716337.1 | Lebiinae | Lebiini | *Dromius angustus* | Jiménez-García et al, 2023 |
| KX087304.1 | Lebiinae | Lebiini | *Lebia chlorocephala* | Direct Submission |
| MH789731.1 | Lebiinae | Lebiini | *Lebiini* sp. 2 ACP-2013 | Crampton-Platt et al, 2015 |
| OQ716374.1 | Lebiinae | Lebiini | *Paradromius* sp. | Jiménez-García et al, 2023 |
| OQ716376.1 | Lebiinae | Lebiini | *Philorhizus* sp. | Jiménez-García et al, 2023 |
| MT554376.1 | Licininae | Chlaeniini | *Chlaenius bioculatus#* | Direct Submission |
| NC_086655.1 | Licininae | Chlaeniini | *Chlaenius naeviger* | Direct Submission |
| OR536810.1 | Licininae | Chlaeniini | *Chlaenius rufifemoratus bimaculatus#* | Li et al, 2024 |
| MN995217 | Licininae | Licinini | *Diplocheila zealandica#* | Fang, 2020 |
| NC_067050.1 | Licininae | Licinini | *Diplocheila zeelandica* | Lin et al, 2024 |
| MT872702.1 | Loricerinae | Loricerini | *Loricera pilicornis* | Direct Submission |
| OQ716355.1 | Nebriinae | Nebriini | *Leistus nubivagus* | Jiménez-García et al, 2023 |
| OW121814 | Nebriinae | Nebriini | *Leistus spinibarbis** | Direct Submission |
| KT876906.1 | Nebriinae | Nebriini | *Nebria brevicollis* | Linard et al, 2016 |
| OX122912 | Nebriinae | Nebriini | *Nebria brevicollis** | Direct Submission |
| MW244066.1 | Nebriinae | Nebriini | *Nebria ingens riversi* | Weng et al, 2021 |
| OX063289 | Nebriinae | Nebriini | *Nebria salina** | Direct Submission |
| NC_064369.1 | Nebriinae | Notiophilini | *Notiophilus quadripunctatus* | Raupach et al, 2022 |
| NC_064368.1 | Omophroninae | Omophronini | *Omophron limbatum* | Raupach et al, 2022 |
| JX412738.1 | Panagaeinae | Panagaeini | *Craspedophorus nobilis* | Timmermans et al, 2015 |
| MF497817.1 | Paussinae | Metriini | *Metrius contractus* | López-López & Vogler 2017 |
| OX384048 | Platyninae | Platynini | *Agonum fuliginosum** | Direct Submission |
| NC_072157.1 | Platyninae | Platynini | *Dyscolus arauzae* | Murienne et al, 2022 |
| NC_072158.1 | Platyninae | Platynini | *Dyscolus diopsis* | Murienne et al, 2022 |
| NC_072162.1 | Platyninae | Platynini | *Dyscolus funereus* | Murienne et al, 2022 |
| NC_072160.1 | Platyninae | Platynini | *Dyscolus fusipalpis* | Murienne et al, 2022 |
| NC_072164.1 | Platyninae | Platynini | *Dyscolus irriguus* | Murienne et al, 2022 |
| NC_072156.1 | Platyninae | Platynini | *Dyscolus lucifuga* | Murienne et al, 2022 |
| NC_072163.1 | Platyninae | Platynini | *Dyscolus montivagus* | Murienne et al, 2022 |
| NC_072155.1 | Platyninae | Platynini | *Dyscolus oopteroides* | Murienne et al, 2022 |
| NC_072154.1 | Platyninae | Platynini | *Dyscolus oreas* | Murienne et al, 2022 |
| NC_072159.1 | Platyninae | Platynini | *Dyscolus orthomus* | Murienne et al, 2022 |
| NC_072161.1 | Platyninae | Platynini | *Dyscolus pollens* | Murienne et al, 2022 |
| NC_072153.1 | Platyninae | Platynini | *Dyscolus rotundiceps* | Murienne et al, 2022 |
| MT554378.1 | Platyninae | Platynini | *Metacolpodes buchanani* | Direct Submission |
| OQ716368.1 | Platyninae | Platynini | *Olisthopus* sp. | Jiménez-García et al, 2023 |
| OQ716310.1 | Platyninae | Sphodrini | *Amaroschema gaudini* | Jiménez-García et al, 2023 |
| OQ716324.1 | Platyninae | Sphodrini | *Calathidius* sp. | Jiménez-García et al, 2023 |
| OQ716325.1 | Platyninae | Sphodrini | *Calathus gomerensis* | Jiménez-García et al, 2023 |
| OQ716349.1 | Platyninae | Sphodrini | *Laemostenus complanatus* | Jiménez-García et al, 2023 |
| NC_085183.1 | Platyninae | Sphodrini | *Synuchus nitidus* | Kim et al, 2024 |
| OQ716375.1 | Pterostichinae | Sphodrini | *Paraeutrichopus* sp. | Jiménez-García et al, 2023 |
| JX313665.1 | Promecognathinae | Promecognathini | *Promecognathus crassus* | Timmermans et al, 2015 |
| NC_030592.1 | Pterostichinae | Pterostichini | *Abax parallelepipedus* | Linard et al, 2016 |
| OQ716340.1 | Pterostichinae | Pterostichini | *Eutrichopus gonzalezi* | Jiménez-García et al, 2023 |
| MK692555.1 | Pterostichinae | Pterostichini | *Orthomus* sp. BMNH 1042407 | Andujar et al, 2019 |
| OZ012734 | Pterostichinae | Pterostichini | *Poecilus cupreus** | Direct Submission |
| KT876910.1 | Pterostichinae | Pterostichini | *Pterostichus madidus* | Linard et al, 2016 |
| MT872694.1 | Pterostichinae | Pterostichini | *Pterostichus melanarius* | Direct Submission |
| NC_044760.1 | Pterostichinae | Pterostichini | *Pterostichus niger* | Direct Submission |
| MN122833.1 | Pterostichinae | Pterostichini | *Pterostichus oblongopunctatus* | Direct Submission |
| MT554377.1 | Pterostichinae | Pterostichini | *Pterostichus prattii#* | Direct Submission |
| KX087349.1 | Pterostichinae | Pterostichini | *Stomis pumicatus* | Direct Submission |
| MN335930.1 | Pterostichinae | Zabrini | *Amara aulica#* | Li et al, 2020 |
| NC_036268.1 | Pterostichinae | Zabrini | *Amara communis* | Direct Submission |
| NC_067049.1 | Pterostichinae | Zabrini | *Amara ovata* | Lin et al, 2024 |
| NC_067792.1 | Rhysodinae | Rhysodini | *Omoglymmius wukong* | Direct Submission |
| KX035156.1 | Rhysodinae | Rhysodini | *Rhysodes* sp. BMNH-844233 | Direct Submission |
| NC_067745.1 | Rhysodinae | Rhysodini | *Rhyzodiastes puetzi* | Direct Submission |
| OZ014572 | Scaritinae | Clivinini | *Clivina fossor** | Direct Submission |
| MF497822.1 | Scaritinae | Scaritini | *Scarites buparius* | López-López & Vogler 2017 |
| NC_060736.1 | Scaritinae | Scaritini | *Scarites subterraneus* | Kyndt E & Kyndt J, 2022 |
| NC_086719.1 | Trechinae | Bembidiini | *Bembidion laterale* | Direct Submission |
| KX087242.1 | Trechinae | Bembidiini | *Bembidion varium* | Direct Submission |
| NC_036261.1 | Trechinae | Bembidiini | *Tachyta nana* | Direct Submission |
| OQ716397.1 | Trechinae | Bembidiini | *Typhlocharis* sp. | Jiménez-García et al, 2023 |
| KX087338.1 | Trechinae | Pogonini | *Pogonus iridipennis* | Direct Submission |
| OQ716306.1 | Trechinae | Trechini | *Aepus gracilicornis* | Jiménez-García et al, 2023 |
| NC_011329.1 | Trachypachidae |  | *Trachypachus holmbergi* | Sheffield et al, 2008 |

Note: species names followed by an asterisk (*) are newly annotated in this study, those followed by a double asterisk (**) are newly sequenced and annotated in this study, and those followed by a hash symbol (#) are species that were suspected to be misidentified.

Table S3 Annotation of the six newly sequenced mitogenomes

1. Annotation of the mitogenome of *Harpalus sinicus*

| Name | location | Length | Direction | Intergenic Nucleotides | Start codon | Stop codon |
| --- | --- | --- | --- | --- | --- | --- |
| trnI(gau) | 1-65 | 65 | forward |  |  |  |
| trnQ(uug) | 73-141 | 69 | reverse | 7 |  |  |
| trnM(cau) | 142-211 | 70 | forward | 0 |  |  |
| ND2 | 212-1240 | 1029 | forward | 0 | ATA | TAA |
| trnW(uca) | 1240-1309 | 70 | forward | -1 |  |  |
| trnC(gca) | 1336-1400 | 65 | reverse | 26 |  |  |
| trnY(gua) | 1417-1484 | 68 | reverse | 16 |  |  |
| COX1 | 1477-3018 | 1542 | forward | -8 | ATT | TAA |
| trnL(uaa) | 3021-3085 | 65 | forward | 2 |  |  |
| COX2 | 3087-3767 | 681 | forward | 1 | ATG | TAA |
| trnK(cuu) | 3770-3840 | 71 | forward | 2 |  |  |
| trnD(guc) | 3841-3907 | 67 | forward | 0 |  |  |
| ATP8 | 3908-4069 | 162 | forward | 0 | ATA | TAA |
| ATP6 | 4063-4740 | 678 | forward | -7 | ATG | TAA |
| COX3 | 4758-5546 | 789 | forward | 17 | ATG | TAA |
| trnG(ucc) | 5549-5614 | 66 | forward | 2 |  |  |
| ND3 | 5615-5968 | 354 | forward | 0 | ATT | TAA |
| trnA(ugc) | 5968-6033 | 66 | forward | -1 |  |  |
| trnR(ucg) | 6034-6099 | 66 | forward | 0 |  |  |
| trnN(guu) | 6102-6167 | 66 | forward | 2 |  |  |
| trnS(gcu) | 6168-6234 | 67 | forward | 0 |  |  |
| trnE(uuc) | 6236-6302 | 67 | forward | 1 |  |  |
| trnF(gaa) | 6301-6366 | 66 | reverse | -2 |  |  |
| ND5 | 6366-8095 | 1730 | reverse | -1 | ATT | T |
| trnH(gug) | 8096-8163 | 68 | reverse | 0 |  |  |
| ND4 | 8163-9503 | 1341 | reverse | -1 | ATG | TAA |
| ND4L | 9497-9790 | 294 | reverse | -7 | ATT | TAA |
| trnT(ugu) | 9793-9855 | 63 | forward | 2 |  |  |
| trnP(ugg) | 9856-9920 | 65 | reverse | 0 |  |  |
| ND6 | 9922-10446 | 525 | forward | 1 | ATT | TAA |
| CYTB | 10446-11585 | 1140 | forward | -1 | ATG | TAA |
| trnS(uga) | 11585-11653 | 69 | forward | -1 |  |  |
| ND1 | 11672-12622 | 951 | reverse | 18 | TTG | TAG |
| trnL(uag) | 12624-12686 | 63 | reverse | 1 |  |  |
| l-rRNA | 12687-14000 | 1314 | reverse | 0 |  |  |
| trnV(uac) | 14001-14073 | 73 | reverse | 0 |  |  |
| s-rRNA | 14074-14861 | 788 | reverse | 0 |  |  |
| Control Region | 14862-16573 | 1712 | forward | 0 |  |  |

2. Annotation of the mitogenome of *Harpalus tridens*

| Name | location | Length | Direction | Intergenic Nucleotides | Start codon | Stop codon |
| --- | --- | --- | --- | --- | --- | --- |
| trnI(gau) | 1-65 | 65 | forward |  |  |  |
| trnQ(uug) | 73-141 | 69 | reverse | 7 |  |  |
| trnM(cau) | 141-210 | 70 | forward | -1 |  |  |
| ND2 | 211-1239 | 1029 | forward | 0 | ATA | TAA |
| trnW(uca) | 1239-1308 | 70 | forward | -1 |  |  |
| trnC(gca) | 1335-1399 | 65 | reverse | 26 |  |  |
| trnY(gua) | 1420-1487 | 68 | reverse | 20 |  |  |
| COX1 | 1480-3021 | 1542 | forward | -8 | ATT | TAA |
| trnL(uaa) | 3024-3088 | 65 | forward | 2 |  |  |
| COX2 | 3090-3770 | 681 | forward | 1 | ATG | TAA |
| trnK(cuu) | 3773-3843 | 71 | forward | 2 |  |  |
| trnD(guc) | 3844-3910 | 67 | forward | 0 |  |  |
| ATP8 | 3911-4072 | 162 | forward | 0 | ATA | TAA |
| ATP6 | 4066-4743 | 678 | forward | -7 | ATG | TAA |
| COX3 | 4769-5557 | 789 | forward | 25 | ATG | TAA |
| trnG(ucc) | 5560-5625 | 66 | forward | 2 |  |  |
| ND3 | 5626-5979 | 354 | forward | 0 | ATT | TAA |
| trnA(ugc) | 5979-6044 | 66 | forward | -1 |  |  |
| trnR(ucg) | 6045-6110 | 66 | forward | 0 |  |  |
| trnN(guu) | 6113-6178 | 66 | forward | 2 |  |  |
| trnS(gcu) | 6179-6245 | 67 | forward | 0 |  |  |
| trnE(uuc) | 6247-6314 | 68 | forward | 1 |  |  |
| trnF(gaa) | 6313-6379 | 67 | reverse | -2 |  |  |
| ND5 | 6380-8108 | 1729 | reverse | 0 | ATT | T |
| trnH(gug) | 8109-8176 | 68 | reverse | 0 |  |  |
| ND4 | 8176-9516 | 1341 | reverse | -1 | ATG | TAA |
| ND4L | 9510-9803 | 294 | reverse | -7 | ATT | TAA |
| trnT(ugu) | 9806-9868 | 63 | forward | 2 |  |  |
| trnP(ugg) | 9869-9933 | 65 | reverse | 0 |  |  |
| ND6 | 9935-10459 | 525 | forward | 1 | ATT | TAA |
| CYTB | 10459-11598 | 1140 | forward | -1 | ATG | TAA |
| trnS(uga) | 11598-11664 | 67 | forward | -1 |  |  |
| ND1 | 11683-12633 | 951 | reverse | 18 | TTG | TAG |
| trnL(uag) | 12635-12697 | 63 | reverse | 1 |  |  |
| l-rRNA | 12698-14010 | 1313 | reverse | 0 |  |  |
| trnV(uac) | 14011-14083 | 73 | reverse | 0 |  |  |
| s-rRNA | 14084-14871 | 788 | reverse | 0 |  |  |
| Control Region | 14872-16066 | 1195 | forward | 0 |  |  |

3. Annotation of the mitogenome of *Harpalus indicus*

| Name | location | Length | Direction | Intergenic Nucleotides | Start codon | Stop codon |
| --- | --- | --- | --- | --- | --- | --- |
| trnI(gau) | 1-65 | 65 | forward |  |  |  |
| trnQ(uug) | 73-141 | 69 | reverse | 7 |  |  |
| trnM(cau) | 141-210 | 70 | forward | -1 |  |  |
| ND2 | 211-1239 | 1029 | forward | 0 | ATA | TAA |
| trnW(uca) | 1239-1308 | 70 | forward | -1 |  |  |
| trnC(gca) | 1335-1399 | 65 | reverse | 26 |  |  |
| trnY(gua) | 1414-1480 | 67 | reverse | 14 |  |  |
| COX1 | 1473-3014 | 1542 | forward | -8 | ATT | TAA |
| trnL(uaa) | 3017-3081 | 65 | forward | 2 |  |  |
| COX2 | 3083-3763 | 681 | forward | 1 | ATG | TAA |
| trnK(cuu) | 3766-3836 | 71 | forward | 2 |  |  |
| trnD(guc) | 3837-3903 | 67 | forward | 0 |  |  |
| ATP8 | 3904-4062 | 159 | forward | 0 | ATA | TAA |
| ATP6 | 4056-4733 | 678 | forward | -7 | ATG | TAA |
| COX3 | 4760-5548 | 789 | forward | 26 | ATG | TAA |
| trnG(ucc) | 5551-5616 | 66 | forward | 2 |  |  |
| ND3 | 5617-5970 | 354 | forward | 0 | ATT | TAA |
| trnA(ugc) | 5970-6035 | 66 | forward | -1 |  |  |
| trnR(ucg) | 6036-6101 | 66 | forward | 0 |  |  |
| trnN(guu) | 6105-6171 | 67 | forward | 3 |  |  |
| trnS(gcu) | 6172-6238 | 67 | forward | 0 |  |  |
| trnE(uuc) | 6240-6306 | 67 | forward | 1 |  |  |
| trnF(gaa) | 6305-6370 | 66 | reverse | -2 |  |  |
| ND5 | 6371-8099 | 1729 | reverse | 0 | ATT | T |
| trnH(gug) | 8100-8168 | 69 | reverse | 0 |  |  |
| ND4 | 8168-9511 | 1344 | reverse | -1 | ATG | TAA |
| ND4L | 9512-9795 | 284 | reverse | 0 | ATT | T |
| trnT(ugu) | 9798-9860 | 63 | forward | 2 |  |  |
| trnP(ugg) | 9861-9925 | 65 | reverse | 0 |  |  |
| ND6 | 9927-10451 | 525 | forward | 1 | ATT | TAA |
| CYTB | 10451-11590 | 1140 | forward | -1 | ATG | TAG |
| trnS(uga) | 11589-11654 | 66 | forward | -2 |  |  |
| ND1 | 11673-12623 | 951 | reverse | 18 | TTG | TAG |
| trnL(uag) | 12625-12687 | 63 | reverse | 1 |  |  |
| l-rRNA | 12688-14000 | 1313 | reverse | 0 |  |  |
| trnV(uac) | 14001-14073 | 73 | reverse | 0 |  |  |
| s-rRNA | 14074-14862 | 789 | reverse | 0 |  |  |
| Control Region | 14863-16612 | 1750 | forward | 0 |  |  |

4. Annotation of the mitogenome of *Harpalus tinctulus*

| Name | location | Length | Direction | Intergenic Nucleotides | Start codon | Stop codon |
| --- | --- | --- | --- | --- | --- | --- |
| trnI(gau) | 1-66 | 66 | forward |  |  |  |
| trnQ(uug) | 70-138 | 69 | reverse | 3 |  |  |
| trnM(cau) | 138-207 | 70 | forward | -1 |  |  |
| ND2 | 208-1236 | 1029 | forward | 0 | ATA | TAA |
| trnW(uca) | 1236-1306 | 71 | forward | -1 |  |  |
| trnC(gca) | 1333-1396 | 64 | reverse | 26 |  |  |
| trnY(gua) | 1410-1477 | 68 | reverse | 13 |  |  |
| COX1 | 1470-3011 | 1542 | forward | -8 | ATT | TAA |
| trnL(uaa) | 3014-3078 | 65 | forward | 2 |  |  |
| COX2 | 3080-3760 | 681 | forward | 1 | ATG | TAA |
| trnK(cuu) | 3767-3837 | 71 | forward | 6 |  |  |
| trnD(guc) | 3838-3903 | 66 | forward | 0 |  |  |
| ATP8 | 3904-4065 | 162 | forward | 0 | ATT | TAA |
| ATP6 | 4059-4736 | 678 | forward | -7 | ATG | TAA |
| COX3 | 4743-5531 | 789 | forward | 6 | ATG | TAA |
| trnG(ucc) | 5534-5599 | 66 | forward | 2 |  |  |
| ND3 | 5600-5953 | 354 | forward | 0 | ATT | TAA |
| trnA(ugc) | 5953-6017 | 65 | forward | -1 |  |  |
| trnR(ucg) | 6018-6085 | 68 | forward | 0 |  |  |
| trnN(guu) | 6088-6152 | 65 | forward | 2 |  |  |
| trnS(gcu) | 6153-6219 | 67 | forward | 0 |  |  |
| trnE(uuc) | 6221-6288 | 68 | forward | 1 |  |  |
| trnF(gaa) | 6287-6351 | 65 | reverse | -2 |  |  |
| ND5 | 6352-8080 | 1729 | reverse | 0 | ATT | T |
| trnH(gug) | 8081-8148 | 68 | reverse | 0 |  |  |
| ND4 | 8148-9488 | 1341 | reverse | -1 | ATG | TAA |
| ND4L | 9482-9775 | 294 | reverse | -7 | ATT | TAA |
| trnT(ugu) | 9778-9841 | 64 | forward | 2 |  |  |
| trnP(ugg) | 9842-9907 | 66 | reverse | 0 |  |  |
| ND6 | 9909-10433 | 525 | forward | 1 | ATT | TAA |
| CYTB | 10433-11572 | 1140 | forward | -1 | ATG | TAG |
| trnS(uga) | 11571-11638 | 68 | forward | -2 |  |  |
| ND1 | 11656-12606 | 951 | reverse | 17 | TTG | TAG |
| trnL(uag) | 12608-12670 | 63 | reverse | 1 |  |  |
| l-rRNA | 12671-13988 | 1318 | reverse | 0 |  |  |
| trnV(uac) | 13989-14059 | 71 | reverse | 0 |  |  |
| s-rRNA | 14060-14847 | 788 | reverse | 0 |  |  |
| Control Region | 14848-16405 | 1558 | forward | 0 |  |  |

5. Annotation of the mitogenome of *Harpalus hauserianus*

| Name | location | Length | Direction | Intergenic Nucleotides | Start codon | Stop codon |
| --- | --- | --- | --- | --- | --- | --- |
| trnI(gau) | 1-65 | 65 | forward |  |  |  |
| trnQ(uug) | 73-141 | 69 | reverse | 7 |  |  |
| trnM(cau) | 141-210 | 70 | forward | -1 |  |  |
| ND2 | 211-1239 | 1029 | forward | 0 | ATA | TAA |
| trnW(uca) | 1239-1308 | 70 | forward | -1 |  |  |
| trnC(gca) | 1335-1399 | 65 | reverse | 26 |  |  |
| trnY(gua) | 1414-1481 | 68 | reverse | 14 |  |  |
| COX1 | 1474-3015 | 1542 | forward | -8 | ATT | TAA |
| trnL(uaa) | 3018-3082 | 65 | forward | 2 |  |  |
| COX2 | 3084-3764 | 681 | forward | 1 | ATG | TAA |
| trnK(cuu) | 3767-3837 | 71 | forward | 2 |  |  |
| trnD(guc) | 3838-3904 | 67 | forward | 0 |  |  |
| ATP8 | 3905-4066 | 162 | forward | 0 | ATA | TAA |
| ATP6 | 4060-4737 | 678 | forward | -7 | ATG | TAA |
| COX3 | 4763-5551 | 789 | forward | 25 | ATG | TAA |
| trnG(ucc) | 5558-5623 | 66 | forward | 6 |  |  |
| ND3 | 5624-5977 | 354 | forward | 0 | ATT | TAA |
| trnA(ugc) | 5977-6042 | 66 | forward | -1 |  |  |
| trnR(ucg) | 6043-6108 | 66 | forward | 0 |  |  |
| trnN(guu) | 6111-6176 | 66 | forward | 2 |  |  |
| trnS(gcu) | 6177-6243 | 67 | forward | 0 |  |  |
| trnE(uuc) | 6245-6311 | 67 | forward | 1 |  |  |
| trnF(gaa) | 6310-6376 | 67 | reverse | -2 |  |  |
| ND5 | 6377-8105 | 1729 | reverse | 0 | ATT | T |
| trnH(gug) | 8106-8173 | 68 | reverse | 0 |  |  |
| ND4 | 8173-9513 | 1341 | reverse | -1 | ATG | TAA |
| ND4L | 9507-9800 | 294 | reverse | -7 | ATT | TAA |
| trnT(ugu) | 9803-9865 | 63 | forward | 2 |  |  |
| trnP(ugg) | 9866-9930 | 65 | reverse | 0 |  |  |
| ND6 | 9932-10456 | 525 | forward | 1 | ATT | TAA |
| CYTB | 10456-11595 | 1140 | forward | -1 | ATG | TAA |
| trnS(uga) | 11595-11661 | 67 | forward | -1 |  |  |
| ND1 | 11680-12630 | 951 | reverse | 18 | TTG | TAG |
| trnL(uag) | 12632-12694 | 63 | reverse | 1 |  |  |
| l-rRNA | 12695-14007 | 1313 | reverse | 0 |  |  |
| trnV(uac) | 14008-14080 | 73 | reverse | 0 |  |  |
| s-rRNA | 14081-14868 | 788 | reverse | 0 |  |  |
| Control Region | 14869-16983 | 2115 | forward | 0 |  |  |

6. Annotation of the mitogenome of *Harpalus pseudotinctulus*

| Name | location | Length | Direction | Intergenic Nucleotides | Start codon | Stop codon |
| --- | --- | --- | --- | --- | --- | --- |
| trnI(gau) | 1-66 | 66 | forward |  |  |  |
| trnQ(uug) | 70-138 | 69 | reverse | 3 |  |  |
| trnM(cau) | 138-207 | 70 | forward | -1 |  |  |
| ND2 | 208-1236 | 1029 | forward | 0 | ATA | TAA |
| trnW(uca) | 1237-1310 | 74 | forward | 0 |  |  |
| trnC(gca) | 1337-1400 | 64 | reverse | 26 |  |  |
| trnY(gua) | 1412-1479 | 68 | reverse | 11 |  |  |
| COX1 | 1472-3013 | 1542 | forward | -8 | ATT | TAA |
| trnL(uaa) | 3016-3079 | 64 | forward | 2 |  |  |
| COX2 | 3081-3764 | 684 | forward | 1 | ATG | TAA |
| trnK(cuu) | 3771-3841 | 71 | forward | 6 |  |  |
| trnD(guc) | 3842-3908 | 67 | forward | 0 |  |  |
| ATP8 | 3909-4070 | 162 | forward | 0 | ATT | TAA |
| ATP6 | 4064-4741 | 678 | forward | -7 | ATG | TAA |
| COX3 | 4748-5536 | 789 | forward | 6 | ATG | TAA |
| trnG(ucc) | 5539-5604 | 66 | forward | 2 |  |  |
| ND3 | 5605-5958 | 354 | forward | 0 | ATC | TAA |
| trnA(ugc) | 5958-6023 | 66 | forward | -1 |  |  |
| trnR(ucg) | 6024-6091 | 68 | forward | 0 |  |  |
| trnN(guu) | 6094-6158 | 65 | forward | 2 |  |  |
| trnS(gcu) | 6159-6225 | 67 | forward | 0 |  |  |
| trnE(uuc) | 6227-6295 | 69 | forward | 1 |  |  |
| trnF(gaa) | 6294-6360 | 67 | reverse | -2 |  |  |
| ND5 | 6361-8089 | 1729 | reverse | 0 | ATT | T |
| trnH(gug) | 8090-8157 | 68 | reverse | 0 |  |  |
| ND4 | 8157-9497 | 1341 | reverse | -1 | ATG | TAA |
| ND4L | 9491-9775 | 285 | reverse | -7 | ATA | TAA |
| trnT(ugu) | 9787-9850 | 64 | forward | 11 |  |  |
| trnP(ugg) | 9851-9917 | 67 | reverse | 0 |  |  |
| ND6 | 9919-10443 | 525 | forward | 1 | ATT | TAA |
| CYTB | 10443-11582 | 1140 | forward | -1 | ATG | TAG |
| trnS(uga) | 11581-11648 | 68 | forward | -2 |  |  |
| ND1 | 11666-12616 | 951 | reverse | 17 | TTG | TAG |
| trnL(uag) | 12618-12680 | 63 | reverse | 1 |  |  |
| l-rRNA | 12681-13997 | 1317 | reverse | 0 |  |  |
| trnV(uac) | 13998-14069 | 72 | reverse | 0 |  |  |
| s-rRNA | 14070-14856 | 787 | reverse | 0 |  |  |
| Control Region | 14857-16266 | 1410 | forward | 0 |  |  |

Table S4 Base composition and skewness of the six newly sequenced mitogenomes

1. Base composition and skewness of the *Harpalus sinicus* mitogenome

| Dataset | Size(bp) | A% | C% | G% | T% | A+T% | AT-Skew | GC-Skew |
| --- | --- | --- | --- | --- | --- | --- | --- | --- |
| Whole genome | 16573 | 41.6% | 11.6% | 8.1% | 38.6% | 80.2% | 0.037 | -0.178 |
| PCGs | 11216 | 34.1% | 10.4% | 11.2% | 44.3% | 78.4% | -0.130 | 0.037 |
| tRNA genes | 1475 | 40.1% | 8.4% | 11.9% | 39.6% | 79.7% | 0.006 | 0.172 |
| rRNA genes | 2102 | 38.8% | 5.9% | 11.7% | 43.6% | 82.4% | -0.058 | 0.330 |
| Control Region | 1712 | 47.8% | 7.2% | 3.6% | 41.4% | 89.2% | 0.072 | -0.333 |

2. Base composition and skewness of the *Harpalus tridens* mitogenome

| Dataset | Size(bp) | A% | C% | G% | T% | A+T% | AT-Skew | GC-Skew |
| --- | --- | --- | --- | --- | --- | --- | --- | --- |
| Whole genome | 16066 | 41.3% | 12.0% | 8.4% | 38.3% | 79.6% | 0.038 | -0.176 |
| PCGs | 11215 | 34.0% | 10.6% | 11.3% | 44.0% | 78.0% | -0.128 | 0.032 |
| tRNA genes | 1475 | 40.1% | 8.4% | 12.1% | 39.4% | 79.5% | 0.009 | 0.180 |
| rRNA genes | 2101 | 39.1% | 6.0% | 11.7% | 43.3% | 82.4% | -0.051 | 0.322 |
| Control Region | 1195 | 47.0% | 8.0% | 4.2% | 40.8% | 87.8% | 0.071 | -0.311 |

3. Base composition and skewness of the *Harpalus indicus* mitogenome

| Dataset | Size(bp) | A% | C% | G% | T% | A+T% | AT-Skew | GC-Skew |
| --- | --- | --- | --- | --- | --- | --- | --- | --- |
| Whole genome | 16612 | 41.7% | 11.8% | 7.9% | 38.6% | 80.3% | 0.039 | -0.198 |
| PCGs | 11205 | 34.0% | 10.5% | 11.1% | 44.3% | 78.3% | -0.132 | 0.028 |
| tRNA genes | 1473 | 40.9% | 8.1% | 11.5% | 39.4% | 80.3% | 0.019 | 0.173 |
| rRNA genes | 2102 | 38.7% | 5.7% | 12.0% | 43.6% | 82.3% | -0.060 | 0.356 |
| Control Region | 1750 | 48.3% | 7.1% | 2.9% | 41.7% | 90.0% | 0.073 | -0.420 |

4. Base composition and skewness of the *Harpalus tinctulus* mitogenome

| Dataset | Size(bp) | A% | C% | G% | T% | A+T% | AT-Skew | GC-Skew |
| --- | --- | --- | --- | --- | --- | --- | --- | --- |
| Whole genome | 16405 | 41.4% | 11.3% | 8.3% | 39.0% | 80.4% | 0.030 | -0.153 |
| PCGs | 11215 | 34.1% | 10.1% | 10.9% | 44.9% | 79.0% | -0.137 | 0.038 |
| tRNA genes | 1483 | 40.7% | 8.5% | 11.6% | 39.2% | 79.9% | 0.019 | 0.154 |
| rRNA genes | 2104 | 38.8% | 5.9% | 11.9% | 43.3% | 82.1% | -0.055 | 0.337 |
| Control Region | 1410 | 46.3% | 7.8% | 4.6% | 41.3% | 87.6% | 0.057 | -0.258 |

5. Base composition and skewness of the *Harpalus hauserianus* mitogenome

| Dataset | Size(bp) | A% | C% | G% | T% | A+T% | AT-Skew | GC-Skew |
| --- | --- | --- | --- | --- | --- | --- | --- | --- |
| Whole genome | 16983 | 41.7% | 11.5% | 8.0% | 38.7% | 80.4% | 0.037 | -0.179 |
| PCGs | 11215 | 34.1% | 10.5% | 11.2% | 44.2% | 78.3% | -0.129 | 0.032 |
| tRNA genes | 1474 | 40.4% | 8.4% | 11.8% | 39.3% | 79.7% | 0.014 | 0.168 |
| rRNA genes | 2101 | 38.8% | 5.9% | 11.8% | 43.4% | 82.2% | -0.056 | 0.333 |
| Control Region | 2115 | 47.4% | 7.4% | 3.8% | 41.4% | 88.8% | 0.068 | -0.321 |

6. Base composition and skewness of the *Harpalus pseudotinctulus* mitogenome

| Dataset | Size(bp) | A% | C% | G% | T% | A+T% | AT-Skew | GC-Skew |
| --- | --- | --- | --- | --- | --- | --- | --- | --- |
| Whole genome | 16266 | 41.3% | 11.6% | 8.3% | 38.8% | 80.1% | 0.031 | -0.166 |
| PCGs | 11209 | 33.9% | 10.1% | 11.1% | 44.8% | 78.7% | -0.139 | 0.047 |
| tRNA genes | 1483 | 40.5% | 9.0% | 11.6% | 38.9% | 79.4% | 0.020 | 0.126 |
| rRNA genes | 2104 | 38.7% | 5.9% | 12.0% | 43.4% | 82.1% | -0.057 | 0.341 |
| Control Region | 1410 | 46.6% | 8.2% | 4.4% | 40.8% | 87.4% | 0.066 | -0.302 |


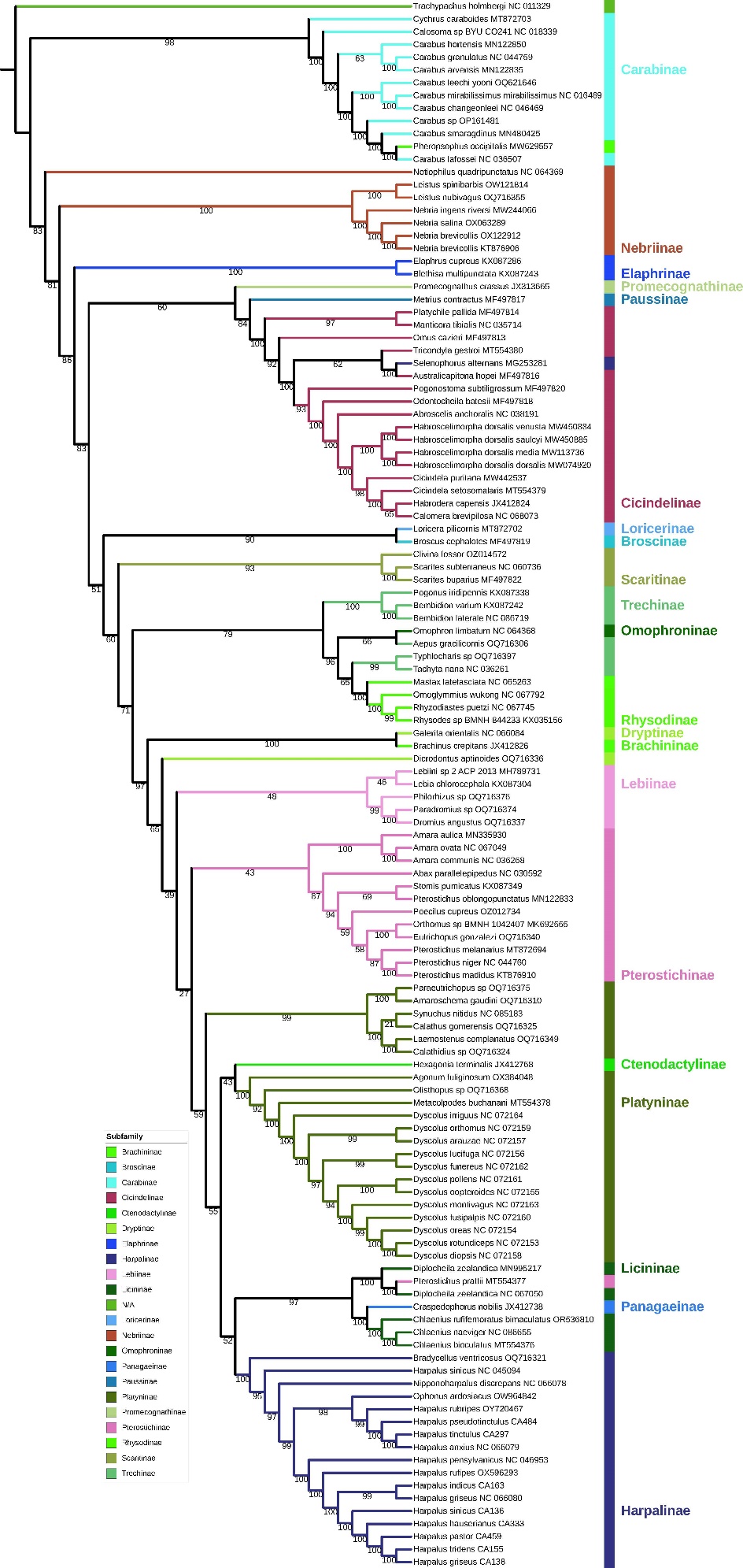


Figure S1 The maximum likelihood tree using the amino acid sequence of PCGs (AA dataset) with full partition (FP).


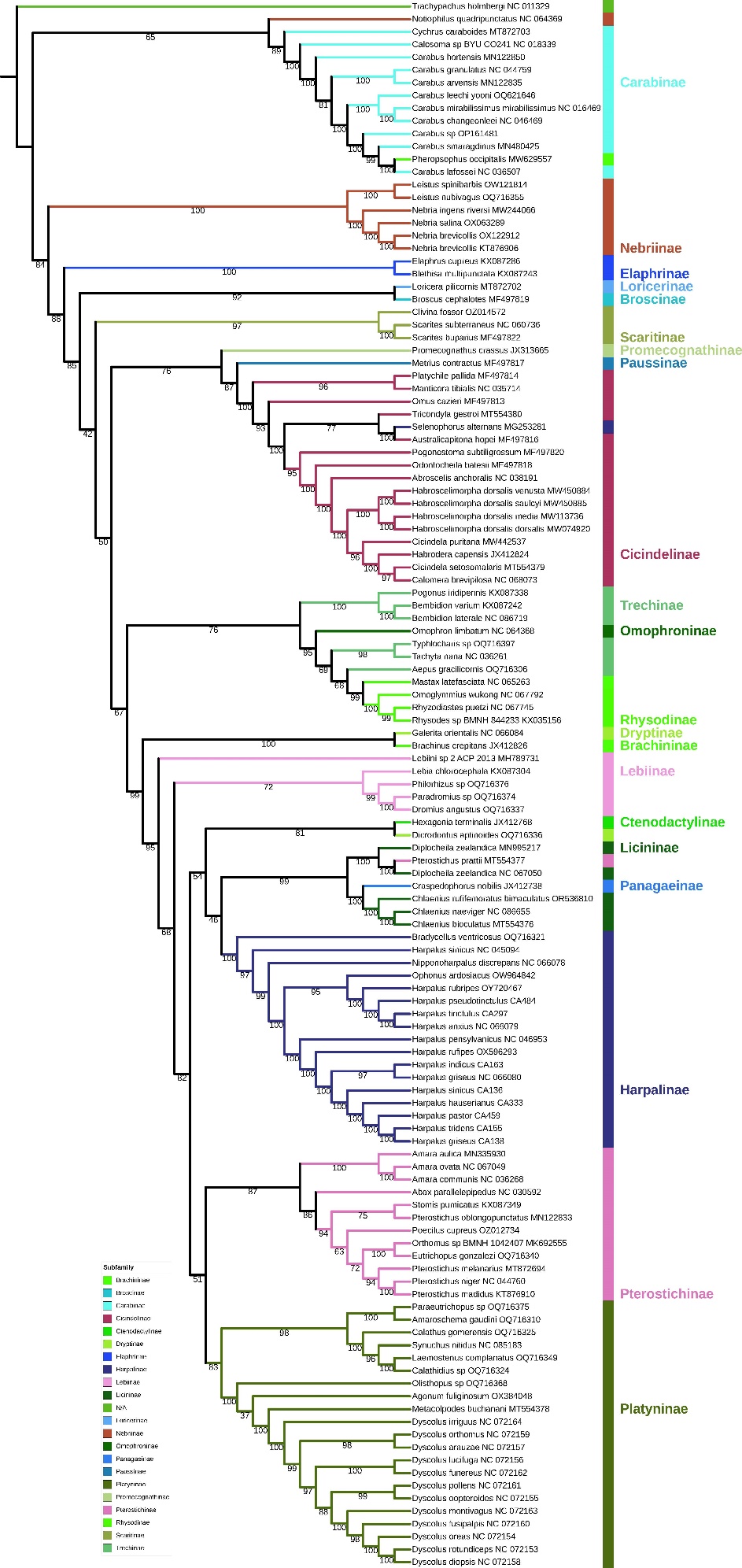


Figure S2 The maximum likelihood tree using the amino acid sequence of PCGs (AA dataset) with merged partition (MP).


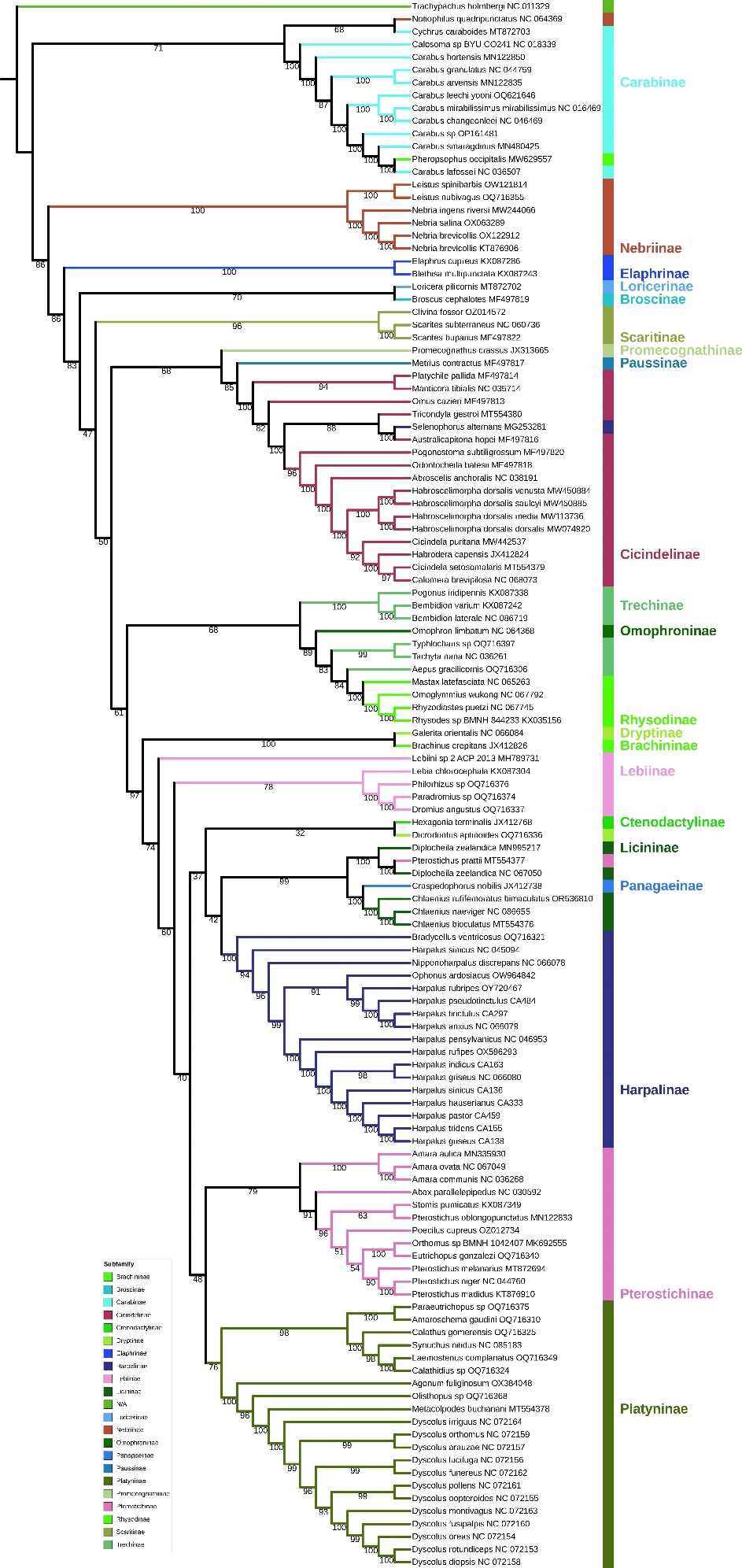


Figure S3 The maximum likelihood tree using the amino acid sequence of PCGs (AA dataset) with no partition (NP).


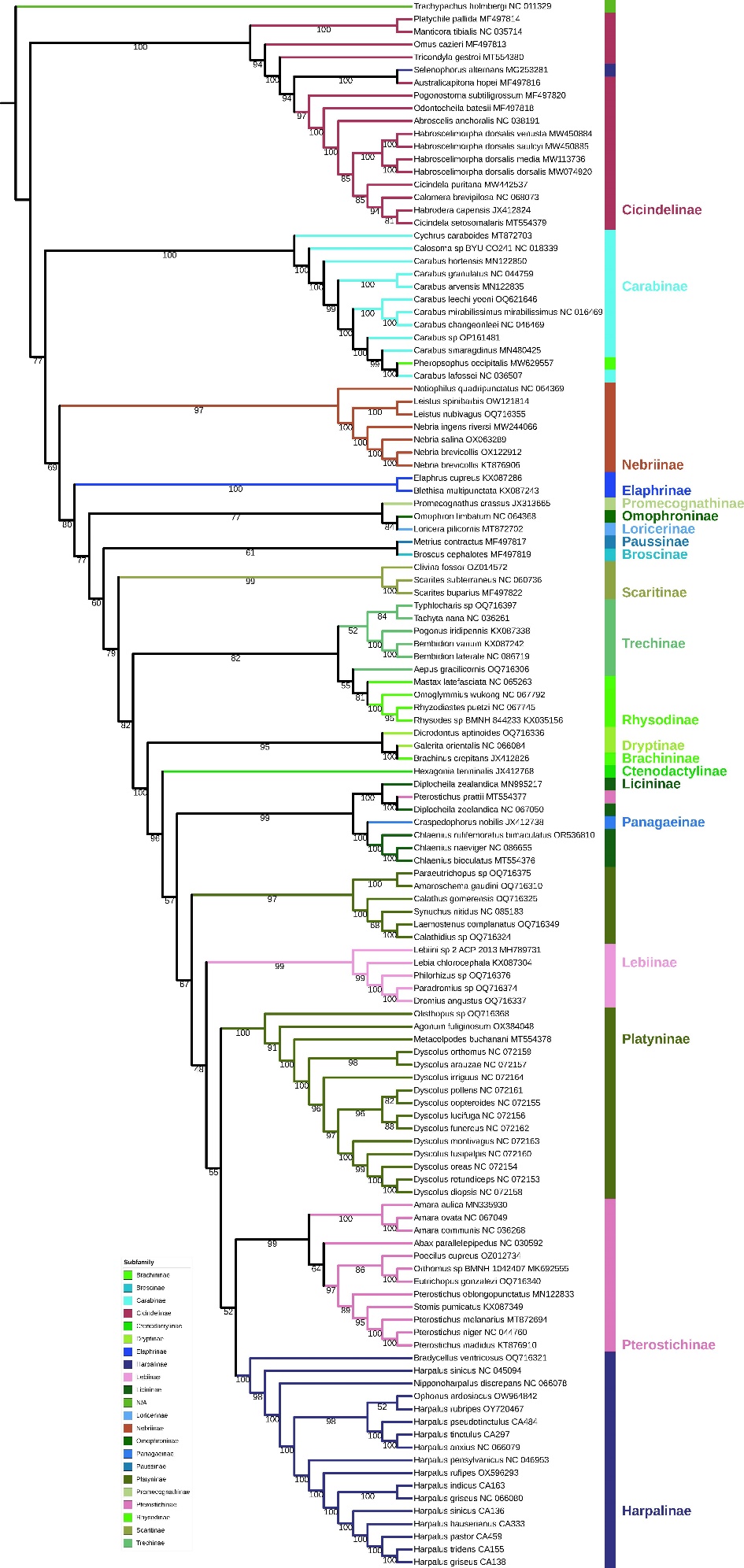


Figure S4 The maximum likelihood tree using the PCG sequences with the third codon position excluded and rRNA gene sequences (P12R dataset) with full partition (FP).


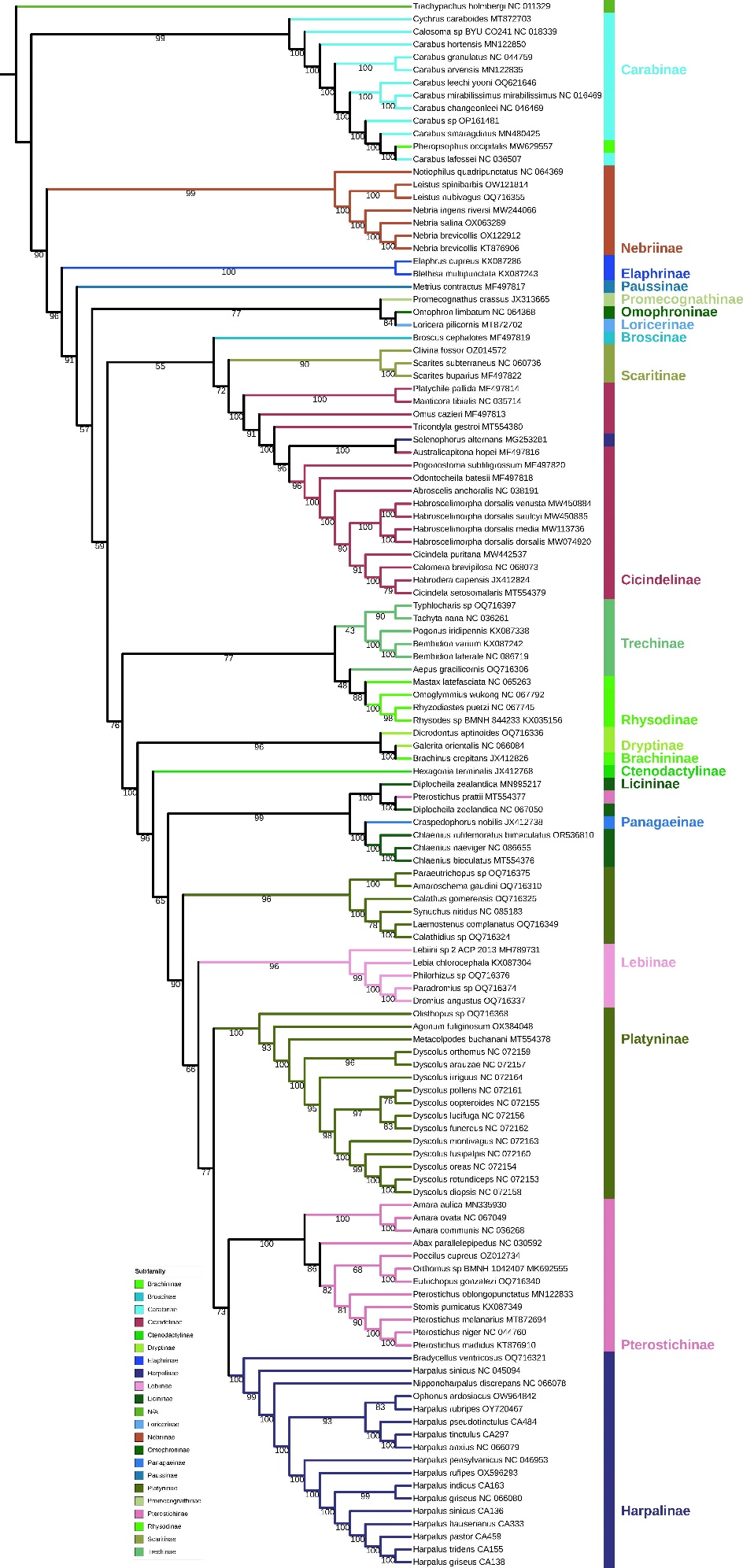


Figure S5 The maximum likelihood tree using the PCG sequences with the third codon position excluded and rRNA gene sequences (P12R dataset) with merged partition (MP).


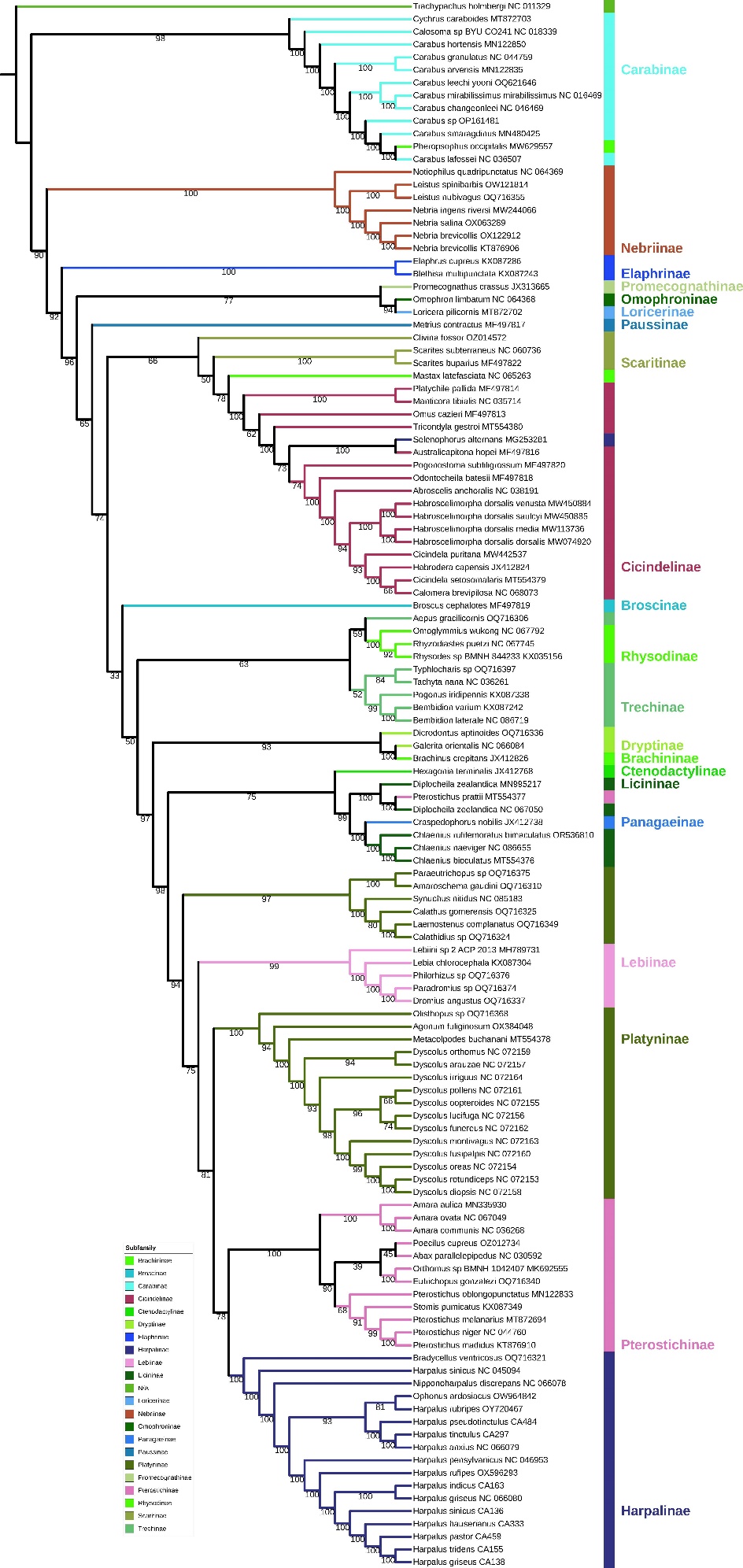


Figure S6 The maximum likelihood tree using the PCG sequences with the third codon position excluded and rRNA gene sequences (P12R dataset) with no partition (NP).


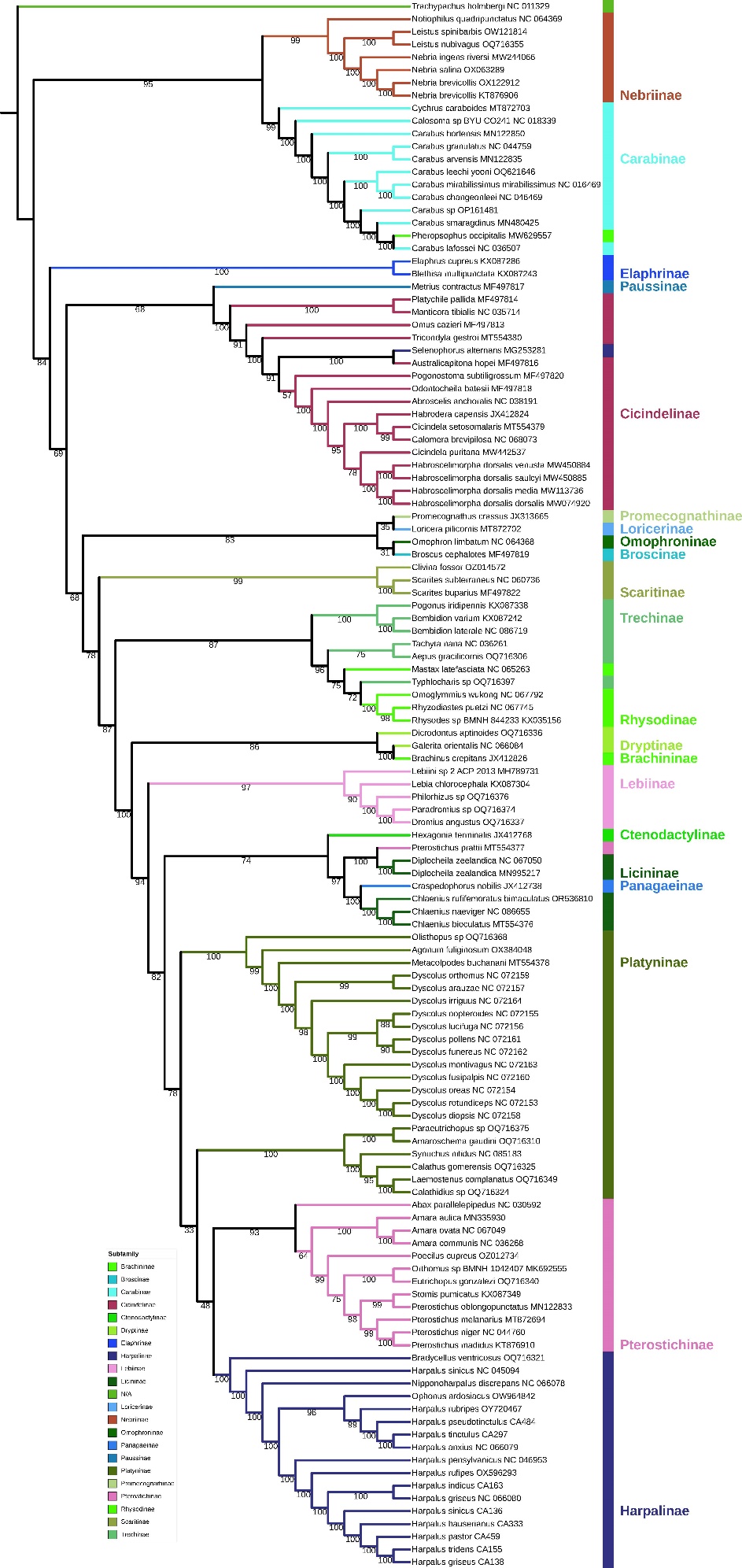


Figure S7 The maximum likelihood tree using the PCG sequences and rRNA gene sequences (P123R dataset) with full partition (FP).


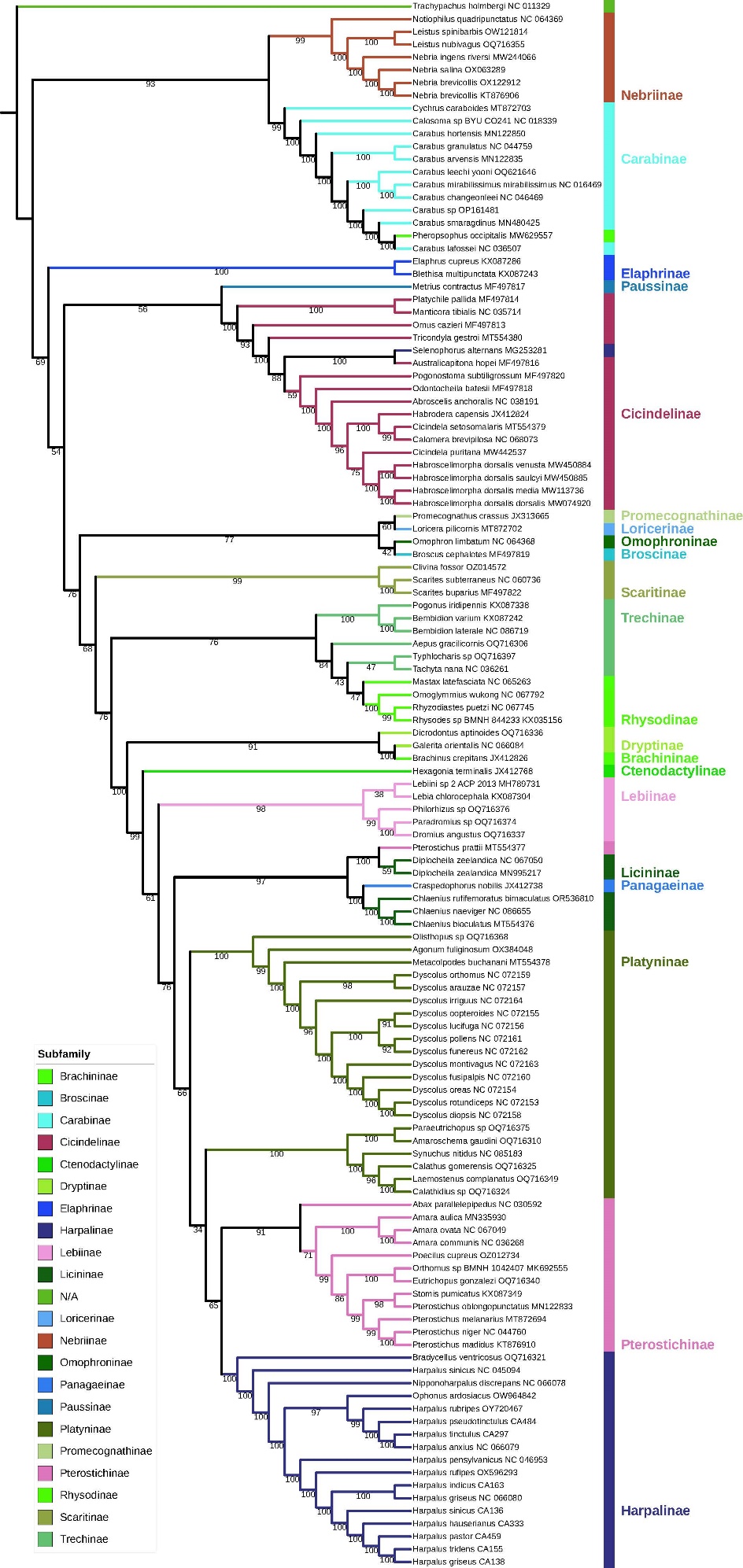


Figure S8 The maximum likelihood tree using the PCG sequences and rRNA gene sequences (P123R dataset) with merged partition (MP).


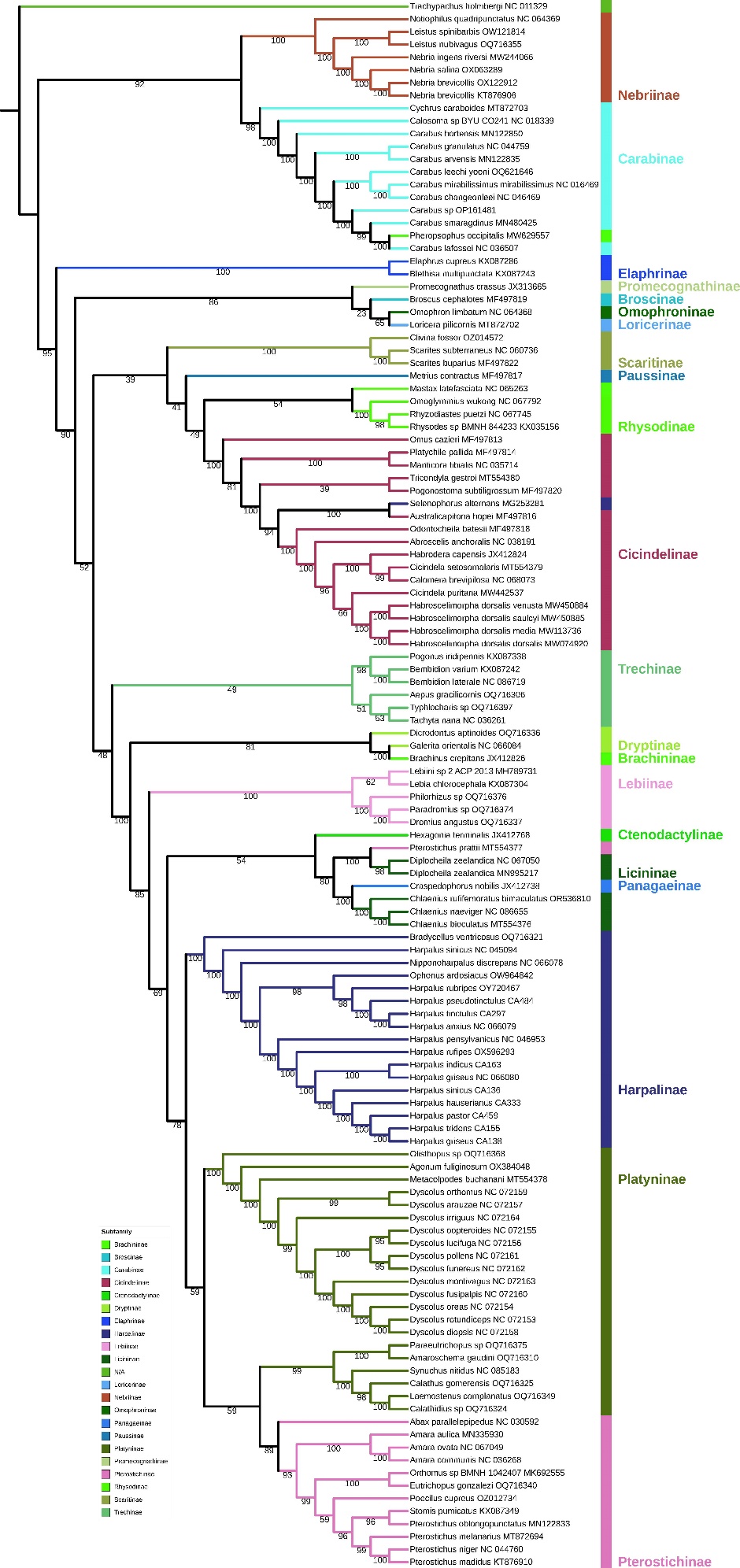


Figure S9 The maximum likelihood tree using the PCG sequences and rRNA gene sequences (P123R dataset) with no partition (NP).


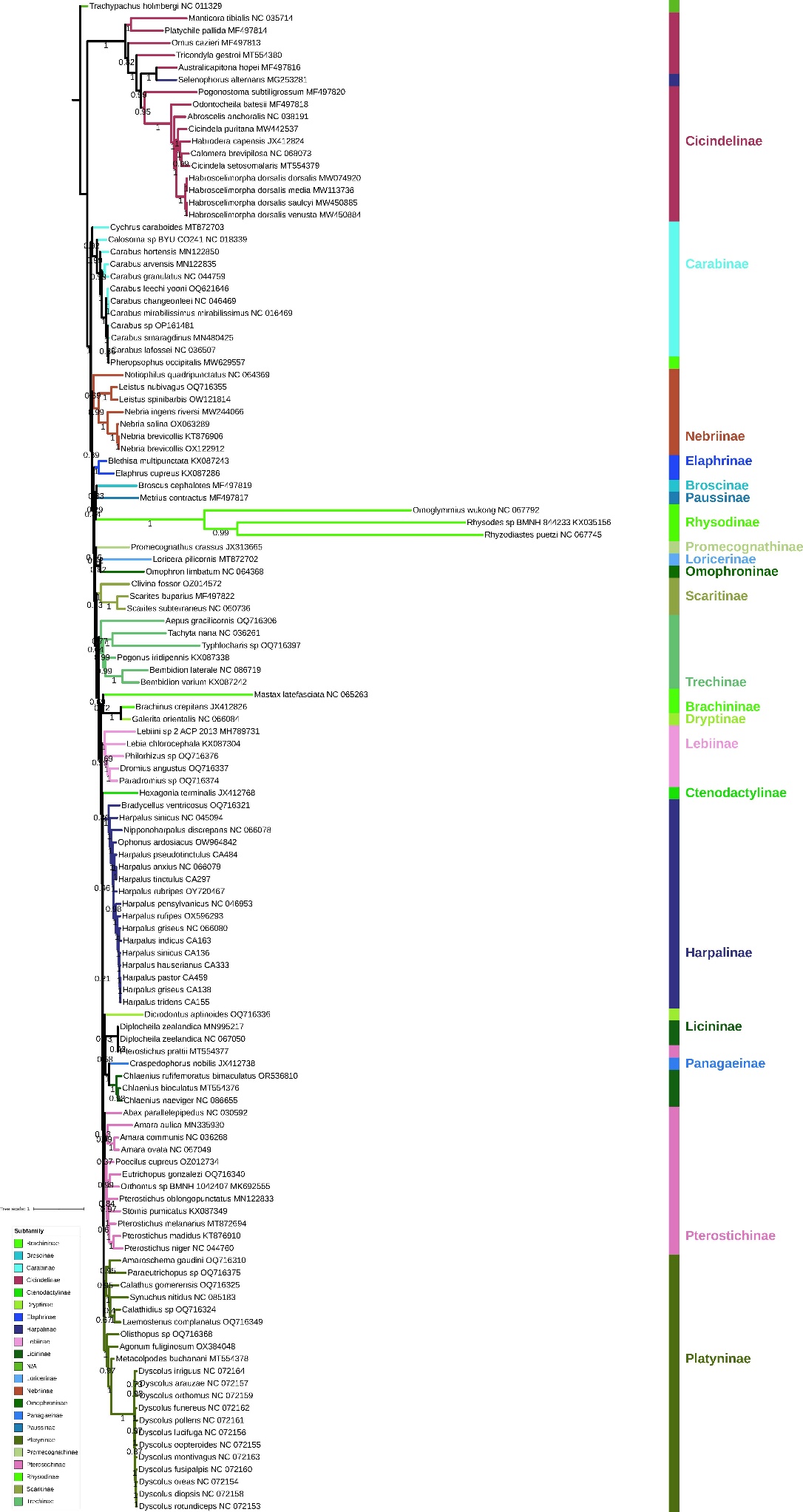


Figure S10 Phylogenetic trees of Carabidae inferred using Bayesian inference with branch lengths.


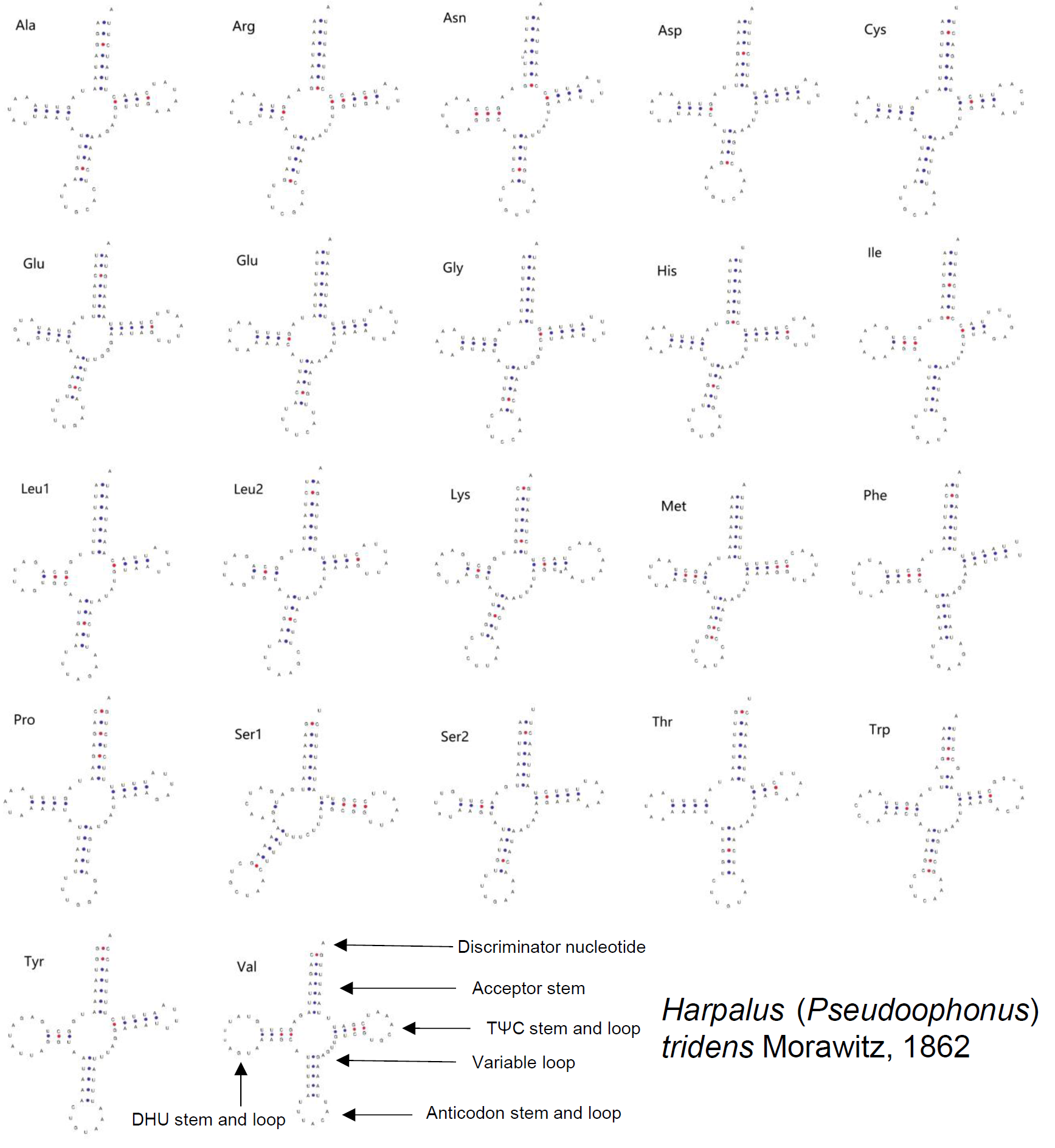


Figure S11. Secondary structure of tRNAs identified in the *Harpalus tridens*.
